# Iron-Based Layered Perovskite Oxyfluoride Electrocatalyst for Oxygen Evolution: Insights from Crystal Facets with Heteroanionic Coordination

**DOI:** 10.1021/jacs.4c05740

**Published:** 2024-11-13

**Authors:** Ryusuke Mizuochi, Yuuki Sugawara, Kengo Oka, Yoshiyuki Inaguma, Shunsuke Nozawa, Toshiyuki Yokoi, Takeo Yamaguchi, Kazuhiko Maeda

**Affiliations:** † Department of Chemistry, School of Science, Institute of Science Tokyo, 2-12-1-NE-2 Ookayama, Meguro-ku, Tokyo 152-8550, Japan; ‡ Institute of Integrated Research, Institute of Science Tokyo, 4259 Nagatsuta-cho, Midori-ku, Yokohama, Kanagawa 226-8501, Japan; § Department of Applied Chemistry, Faculty of Science and Engineering, Kindai University, 3-4-1 Kowakae, Higashi-osaka, Osaka 577-8502, Japan; ∥ Department of Chemistry, Faculty of Science, Gakushuin University, 1-5-1 Mejiro, Toshima-ku, Tokyo 171-8588, Japan; ⊥ Institute of Materials Structure Science, High Energy Accelerator Research Organization, 1-1 Oho, Tsukuba, Ibaraki 305-0801, Japan; # Research Center for Autonomous Systems Materialogy (ASMat), Institute of Science Tokyo, 4259 Nagatsuta-cho, Midori-ku, Yokohama, Kanagawa 226-8501, Japan

## Abstract

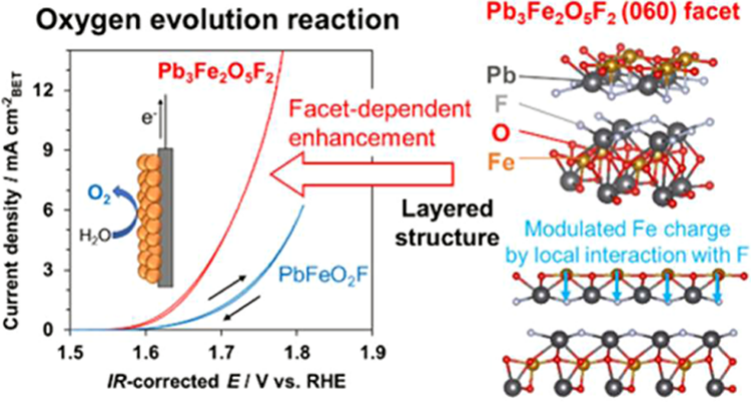

Mixed-anion compounds have recently attracted attention as solid-state materials that exhibit properties unattainable with those of their single-anion counterparts. However, the use of mixed-anion compounds to control the morphology and engineer the crystal facets of electrocatalysts has been limited because their synthesis method is still immature. This study explored the electrocatalytic properties of a Pb–Fe oxyfluoride, Pb_3_Fe_2_O_5_F_2_, with a layered perovskite structure for oxygen evolution reaction (OER) and compared its properties in detail with those of a bulk-type cubic three-dimensional (3D) perovskite, PbFeO_2_F. A Pb_3_Fe_2_O_5_F_2_ electrode prepared with carbon nanotubes and a graphite sheet as a conductive support and a substrate, respectively, demonstrated better OER performance than a PbFeO_2_F electrode. The role of specific crystal facets of Pb_3_Fe_2_O_5_F_2_ in enhancing the OER activity was elucidated through electrochemical analysis. Density functional theory calculations indicated that the Pb_3_Fe_2_O_5_F_2_ (060) facet with Fe sites exhibited a lower theoretical overpotential for the OER, which was attributed to a moderately strong interaction between the active sites and the reaction intermediates; this interaction was reinforced by the strong electron-withdrawing behavior of fluoride ions. This finding offers new insights for developing efficient electrocatalysts based on oxyfluorides, leveraging the high electronegativity of fluorine to optimize the electronic states at active sites for the OER, without relying on precious metals.

## Introduction

Mixed-anion compounds, in which several anionic species are coordinated to a metal center in a single phase,^1,2^ have been used in chemical reactions as photocatalysts,^3,4^ electrocatalysts,^5^ and battery cathode materials.^4,6,7^ In each of these applications, the incorporation of heteroanions into a material that was originally a single-anion compound enhances its photocatalytic or electrochemical performance by modifying the electronic states of the material. For example, in recent years, mixed-anion compounds have been developed as electrocatalysts for oxygen evolution reaction (OER),^5^ which is an important half-reaction for producing hydrogen via electrochemical water-splitting and for charging process in lithium–air next-generation secondary batteries. In mixed-anion electrocatalysts, the OER performance (including stability during operation) has been improved by introducing anions with different electronegativities into a single-anion electrocatalyst. In one study, mixing S^2–^ and OH^–^ to form NiCo_2_(SOH)_*x*_ led to a decreased electron density of antibonding orbitals in the original metal–anion bonding of NiCo_2_S_4_, which in turn led to stabilization of the electrocatalyst while maintaining the OER activity.^8^ In another example, the incorporation of N^3–^ ions into CoS_2_ contributed to lowering its overpotential in both the OER and the hydrogen evolution reaction by changing the adsorption energy of reaction intermediates adsorbed onto the electrocatalyst surface. This change was caused by a lowering of the energy levels of the metal d orbitals in the catalytic Co center.^9^ In both cases, the mixing of anions with different electronegativities differentiated the electronic states of the materials from those of their single-anion counterparts, thereby changing their electrochemical properties. Researchers have thus far designed numerous OER electrocatalysts (mostly metal oxides) by varying and combining cation species to optimize the electronic states.^10,11^ The use of the mixed-anion concept enables the OER activity to be improved from anion-derived parameters, in addition to the cation-derived improvements already introduced. This approach provides an unprecedented opportunity to develop electrocatalysts and expand the selection of electrocatalytic materials.

Regarding synthesis, various methods are available to control the morphology and optimize the electronic state of cation-controlled metal oxide electrocatalysts.^12−15^ These oxides have demonstrated improved OER activity, which often relies on specific crystal facets.^16−18^ Meanwhile, synthesis methods for mixed-anion compounds are still immature, and controlling the morphology of mixed-anion electrocatalysts and engineering their crystal facets are difficult.

Oxyfluorides are mixed-anion compounds that possess O^2–^ and F^–^; they are interesting materials because F exhibits the highest electronegativity among the elements. This high electronegativity leads to unique properties of the oxyfluorides, even compared with those of other mixed-anion compounds,^19−23^ thereby expanding the range of oxyfluoride applications.^24−31^ A Ni–Co oxyfluoride electrocatalyst exhibited relatively high OER performance, which was attributed to incorporated F^–^ ions modifying the surface electronic configuration by strongly withdrawing electrons from active sites as a result of their high electronegativity.^32^ However, a strategy to incorporate F^–^ ions into the OER catalysts to improve their catalytic performance was not specified.

Exploiting F as the most electronegative element enables the electronic (oxidation) states on active sites in materials to be favorable for electrocatalysis. This approach introduces the possibility of designing OER electrocatalysts with high stability and performance even without noble and rare metals such as Ir, Ru, Co, and Ni, which are used in high-performance OER electrocatalysts. However, oxyfluorides in electrocatalysis have been scarcely explored thus far; only 13 studies of such materials have been reported (compared with more than 5000 reports related to oxides).^32−46^ Moreover, the literature contains no reports detailing the effect of introducing F in terms of the oxidation states of the active sites for the OER. In addition, although the crystal facet dependence of the OER activity has been investigated for some oxide electrocatalysts, as previously mentioned,^16−18^ it has never been studied for oxyfluorides. Even among all mixed-anion compounds, an OER electrocatalyst of BaTaO_2_N is the only material whose facet dependence has been studied computationally.^47^ Thus, much room exists for exploring oxyfluoride electrocatalysts.

**Figure 1:**
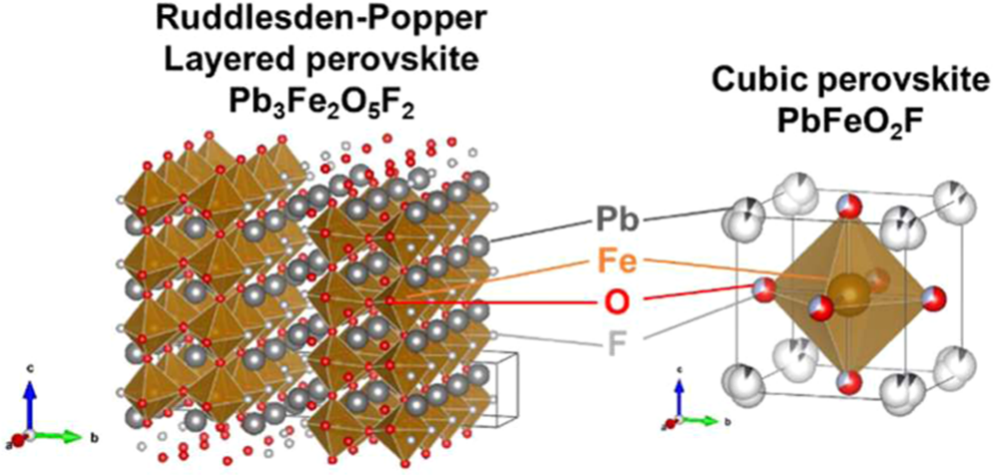
Crystal structures of layered perovskite, Pb_3_Fe_2_O_5_F_2_ (left), in which O^2–^ and F^–^ anions are ordered,^48^ and cubic perovskite, PbFeO_2_F (right), in which O^2–^ and F^–^ anions are disordered.^50^

The present study focuses on a Pb–Fe oxyfluoride, Pb_3_Fe_2_O_5_F_2_ (Figure 1, left),^48^ with a layered perovskite structure as a model precious-metal-free OER electrocatalyst. In a preliminary report, we showed that Pb_3_Fe_2_O_5_F_2_ functions as an OER electrocatalyst^49^ that exhibits superior OER performance compared with the cubic perovskite oxyfluoride PbFeO_2_F (Figure 1, right).^50^ However, the origin of this superior OER activity has not been revealed, and its detailed mechanism of OER remains unclear. Therefore, we investigate here the relationship between the introduction of F and the electrochemical performance toward the OER using a combination of electrochemical measurements and density functional theory (DFT) calculations. The results of the experimental and computational investigation indicate that the growth of the (0*k*0) crystal facets in the stacking direction of the layered structure strongly influences the enhanced OER activity of the Pb_3_Fe_2_O_5_F_2_. In addition, DFT charge analysis of the Fe atoms on the facet demonstrates that the F atoms lining the interlayer lower the electron density of the Fe and modify the oxidation state, making it more favorable for the OER. This finding provides an alternative approach to enhancing the OER activity without using precious metals by incorporating F^–^ ions into a layered structure to withdraw electrons from the active sites.

## Experimental Section

### Synthesis

According to a previous report,^48,49^ Pb_3_Fe_2_O_5_F_2_ powder was synthesized through a solid-state reaction under vacuum. A mixture of PbO (99.9%, Rare Metallic), PbF_2_ (99.9%, Mitsuwa Pure Chemical), and α-Fe_2_O_3_ (99.9%, FUJIFILM Wako Pure Chemical) powder, which were mixed in molar ratios of PbO/PbF_2_/α-Fe_2_O_3_ = 2:1:1, was wrapped in a Pt sheet and then heated at 873 K for 12 h in an evacuated Pyrex tube. In addition, the heating time was changed to 4, 8, and 18 h to confirm the effect of crystal growth in the direction of a particular crystal plane.

PbFeO_2_F powder was synthesized through a solid-state reaction under high pressure using a mixture of PbO (99.9%, Kanto Chemical), PbF_2_ (99.999%, Soekawa Chemical), and Fe_2_O_3_ (99.99%, Rare Metallic).^50^ A stoichiometric mixture of the starting materials was dried under reduced pressure at ∼573 K overnight. The mixture was sealed in an Au capsule (0.2 mm thick, 3.1 mm inner diameter, and 3.2 mm depth), which was placed in a NaCl sleeve. The capsule and sleeve were inserted into a pyrophyllite cube block (one side, 13 mm) equipped with a cylindrical graphite heater. The mixture was allowed to react in a TRY cubic multianvil-type high-pressure apparatus (NAMO 2001) at 6.0 GPa and 1173 K for 30 min and then quenched to room temperature. The production of single-phase PbFeO_2_F and Pb_3_Fe_2_O_5_F_2_ was confirmed by X-ray diffraction (XRD) analysis.

### Characterization

The phase purity of the synthesized samples was confirmed by XRD analysis using a Rigaku MiniFlex600 diffractometer (monochromatized Cu Kα). Scanning electron microscopy (SEM) observations combined with energy-dispersive X-ray spectroscopy (EDS) measurements were conducted on a JEOL JSM-IT100 instrument equipped with a KEYENCE VE-9800 apparatus. Surface electronic states on the samples were examined by X-ray photoelectron spectroscopy (XPS; ESCA-3400, Shimadzu). The binding energies observed by XPS were corrected in reference to the C 1s peak (285.0 eV) for each sample. X-ray absorption fine structure (XAFS) measurements were performed at the beamline BL-9C of the Photon Factory (High Energy Accelerator Research Organization, Tsukuba, Japan) under the approval of the Photon Factory Advisory Committee (Proposal No. 2022G676). The X-ray energy was varied by using a Si(111) double-crystal monochromator. The XAFS data were processed using the Athena software.^51^ The Brunauer–Emmett–Teller (BET) specific surface areas of the samples were determined by N_2_ adsorption–desorption isotherms acquired at 77 K using a BELSORP-max II (MicrotracBEL), which can measure a sample with a specific surface area as small as 0.01 m^2^ g^–1^. Physisorbed water was removed prior to the measurements by heating the samples at 373 K under a vacuum overnight. The specific surface areas were calculated using the BELMaster software provided with the BELSORP-Max II. To examine dissolved metal species in solution after the controlled-potential electrolysis described in the next subsection, analysis by inductively coupled plasma optical emission spectrometry (ICP-OES) was conducted with a 5100 VDV apparatus (Agilent Technologies) at the Materials Analysis Division, Core Facility Center, Institute of Science Tokyo. A fluoride ion assay kit (Ponalkit-F, Fujifilm Wako Pure Chemical) was also used to detect dissolved fluoride ions in solution after the electrolysis.

### Electrode Preparation

Electrode preparation was conducted using a graphite sheet (PGS graphite sheet of EYG type, Panasonic) as a substrate and multiwalled carbon nanotubes (MWCNTs) (90%, Sigma-Aldrich) as a conductive support, as described elsewhere.^52^ A catalyst ink was prepared by sonicating some amount of the oxyfluoride powder and 1 mg of the MWCNTs in a mixture of 0.1 mL of 5 wt % Nafion solution (product 274704, Sigma-Aldrich), 0.3 mL of isopropanol, and 0.6 mL of pure water for 2 h. The ink (36 μL) was drop-casted onto a graphite sheet with a geometric area of 1 cm^2^ and dried thoroughly in air. The loading of oxyfluoride powder onto the graphite sheet was 0.108 and 0.180 mg cm^–2^ for samples prepared with 3 and 5 mg of powder in ink, respectively. The MWCNTs and the graphite sheet are hereafter denoted by “CNT” and “GS”, respectively.

### Electrochemical Measurements

Electrochemical measurements were carried out at room temperature with a potentiostat (HZ-Pro, Hokuto Denko) and an electrochemical cell. The cell was made of Pyrex and was a three-electrode-type system with Pt wire and an Ag/AgCl electrode (saturated KCl aqueous solution) as the counter and reference electrodes, respectively. A 1 M aqueous KOH (>98.0%, Fujifilm Wako Pure Chemical) solution was used as the electrolyte, which was saturated with Ar gas bubbled sufficiently before all the measurements. The electrochemical potentials measured toward the Ag/AgCl reference were converted to those toward the reversible hydrogen electrode (RHE) using the following equation
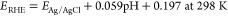


In this work, electrochemical activities are reported as current densities per geometric area of the electrodes or the BET specific surface area of the catalyst powders (denoted by mA cm_geo_^–2^ or mA cm_BET_^–2^, respectively). Before the main measurements, the sample electrodes were pretreated by ten cyclic voltammetry (CV) scans between +0.1 and +1.2 V vs RHE and then 20 CV scans between +1.2 and +2.0 V at a scan rate of 100 mV s^–1^ in Ar-saturated 1 M KOH solution. The main CV scans were then performed five times between +1.2 and +2.0 V at a scan rate of 10 mV s^–1^ in the same solution. Ohmic losses were corrected using the measured current (*I*) and electrolyte resistance (*R*) determined from AC impedance according to the following equation
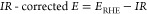


Electrochemical impedance spectroscopy (EIS) was conducted by using the same potentiostat and electrochemical setup. The measurement conditions were set to an AC amplitude of 5 mV and a frequency range of 100 kHz to 0.1 Hz at +1.7 V vs RHE.

### Computational Details in DFT Calculations

All spin-polarized calculations were conducted using the DFT method, as implemented in the Vienna Ab initio Simulation Package (VASP).^53,54^ For the Pb_3_Fe_2_O_5_F_2_ and PbFeO_2_F systems, we carried out the optimization to construct supercells (2 × 2 × 2) with antiferromagnetic properties following the previous studies,^48,50^ using unit cells and atomic positions from the crystallographic information file data reported there. The PbFeO_2_F supercell was manually set up, so that the arrangement of fluorine atoms was random. The core electrons were expressed by the projector-augmented wave,^55^ and the valence electrons were expanded by the plane-wave basis set to a cutoff energy of 500 eV. The Perdew–Burke–Ernzerhof functional was used for the generalized gradient approximation part.^56^ For all of the calculations, the tetrahedron method with Blöchl correction was used in the smearing for the electron occupation near the Fermi level. The convergence thresholds for the electronic state calculation and geometry optimization were set to 1.0 × 10^–4^ eV and 1.0 × 10^–3^ eV Å^–1^ in energy and force, respectively. When the surface calculations were performed for adsorption onto a cut crystal surface with intermediates for water oxidation, the thickness of the vacuum layer over an exposed facet of the oxyfluoride was set to 15 Å to avoid interlayer interactions.

The OER on the oxyfluorides was studied via the four-electron pathway known as the adsorbate evolution mechanism (AEM)^57−59^ or the lattice oxygen-mediated mechanism (LOM).^60−62^ The following explanation is first for the AEM process on basic conditions. In the first step, the active site on the oxyfluoride (denoted as *) takes an OH^–^ ion in the basic solution and releases one electron (e^–^), leading to the formation of adsorbed OH*. Next, another OH^–^ ion takes a proton from the OH* and releases one electron, leaving adsorbed O*. Another OH^–^ ion then participates and reacts with the O* to form adsorbed OOH* with the release of one electron. Finally, OOH* generates O_2_ to pass a proton to another OH^–^ ion with the release of one electron, reverting to * eventually. The four elementary steps of the OER in the basic condition can be described as
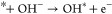
1
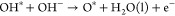
2
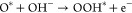
3
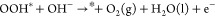
4

To avoid calculating the free energy of the O_2_ gas, we considered the experimental reaction energy of 2H_2_O → O_2_ + 2H_2_ (4.92 eV). In addition, the energy of the OH molecule regarded as (OH^–^ – e^–^) for every step can be replaced by the resulted energy from subtracting one-half of the energy of the H_2_ molecule from that of the H_2_O molecule at 298 K.^63^ Accordingly, the change in the Gibbs free energy in each step can be written as
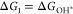
5
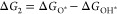
6
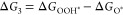
7
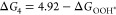
8where Δ*G*_OH*_, Δ*G*_O*_, and Δ*G*_OOH*_ are the Gibbs free energy changes of OH*, O*, and OOH*, which represent the energies associated with adsorption onto the active site (*). These adsorption energies are defined as
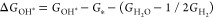
9
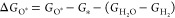
10
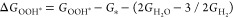
11where G_*_, *G*_OH*_, *G*_O*_, and *G*_OOH*_ are the Gibbs free energies of *, OH*, O*, and *OOH, respectively. *G*_H_2_O_ and *G*_H_2__ are the Gibbs free energies of the H_2_O and H_2_ molecules, respectively. The Gibbs free energy (*G*) can be calculated by
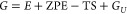
12where *E* denotes the total energy of the system obtained from DFT calculations, and ZPE and TS are the zero-point energy and the entropy correction, respectively. The ZPE and the TS for OH*, O*, *OOH, H_2_O, and H_2_ were calculated based on literature values.^64^ The equation *G*_*U*_ = −*n*_e_*U* is true, where *n*_e_ represents the number of electrons and *U* is the electrode potential. The *G*_*U*_ term includes the dependence of pH as well as the effect of *U*; however, the *G*_*U*_ terms in eq 12 are canceled out when subtracting the formation Gibbs energy for each state to calculate the Gibbs free energy changes of Δ*G*_1_ – Δ*G*_4_. In addition, electron transfer for every step of the AEM process was assumed to have a small and negligible energy barrier. Thus, the OER property was only governed by the free energy changes for each elementary step without the contribution of *G*_*U*_. The step with the greatest energy change for the OER process is defined as the potential-determining step, and the OER overpotential (η_DFT_) is defined as
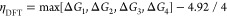
13

A catalyst with lower η_DFT_ exhibits better OER activity, whose interpretation is the same as that of the experimental overpotential in the OER.

On the other hand, DFT calculation for OER through the LOM process was carried out to assume the following four elementary steps, according to the previous report on perovskite oxides, which addressed both the AEM and LOM processes.^62^
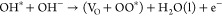
14
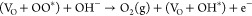
15
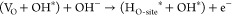
16
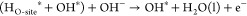
17where OH*, OO*, V_O_, and H_O-site_* are adsorbed OH and OO molecules onto the active metal site, lattice oxygen vacancy on the surface, and an adsorbed H atom onto the lattice oxygen on the surface. In the beginning of eq 14, the state (adsorbed OH onto the active site of the crystal surface) is the same as eq 2 in the AEM process. The DFT calculations of the LOM process from this point onward were performed in the same way as the AEM process, as mentioned above.

## Results and Discussion

### Electrochemical OER Performance of Pb_3_Fe_2_O_5_F_2_

**Figure 2:**
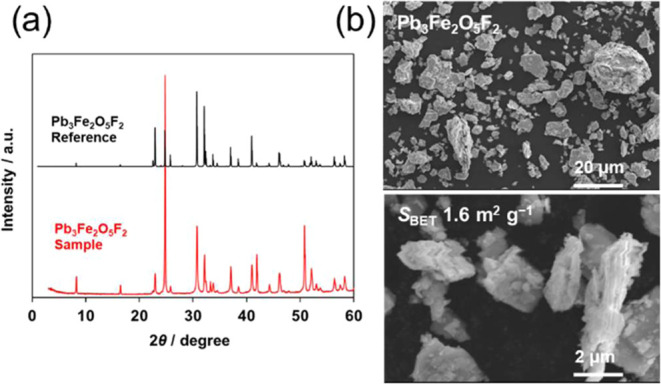
(a) XRD patterns for Pb_3_Fe_2_O_5_F_2_ powder synthesized with a heating time of 12 h and its reference data.^48^ (b) SEM images of synthesized Pb_3_Fe_2_O_5_F_2_.

In accordance with a previous report,^48^ PbO, PbF_2_, and α-Fe_2_O_3_ powders were mixed in a stoichiometric ratio, and the mixture was heated in a vacuum-sealed glass tube at 873 K for 12 h to synthesize the Pb_3_Fe_2_O_5_F_2_ powder sample. XRD analysis confirmed the production of single-phase Pb_3_Fe_2_O_5_F_2_ with a double-layered Ruddlesden–Popper phase (Figure 2a), which has ordered fluoride ions in the structure (Figure 1, left). For comparison, cubic perovskite PbFeO_2_F powder was synthesized through a solid-state reaction under high pressure^50^ and was also confirmed to be a single phase through XRD analysis (Figure S1). It was reported that the PbFeO_2_F structure had disordered fluoride ions (Figure 1, right), as revealed by physicochemical characterization, such as Mössbauer spectroscopy, in the above literature.

The as-synthesized Pb_3_Fe_2_O_5_F_2_ (Figure 2b) and PbFeO_2_F (Figure S2) powders were observed by SEM. The synthesized Pb_3_Fe_2_O_5_F_2_ particles exhibited a relatively large size distribution of 1–20 μm. However, the PbFeO_2_F particles had a smaller size distribution of 5–10 μm. The BET specific surface area (*S*_BET_) was determined to be 1.6 m^2^ g^–1^ for Pb_3_Fe_2_O_5_F_2_ and 3.2 m^2^ g^–1^ for PbFeO_2_F. This difference might reflect the larger size distribution and the average size of Pb_3_Fe_2_O_5_F_2_ particles compared with those of PbFeO_2_F particles.

**Figure 3:**
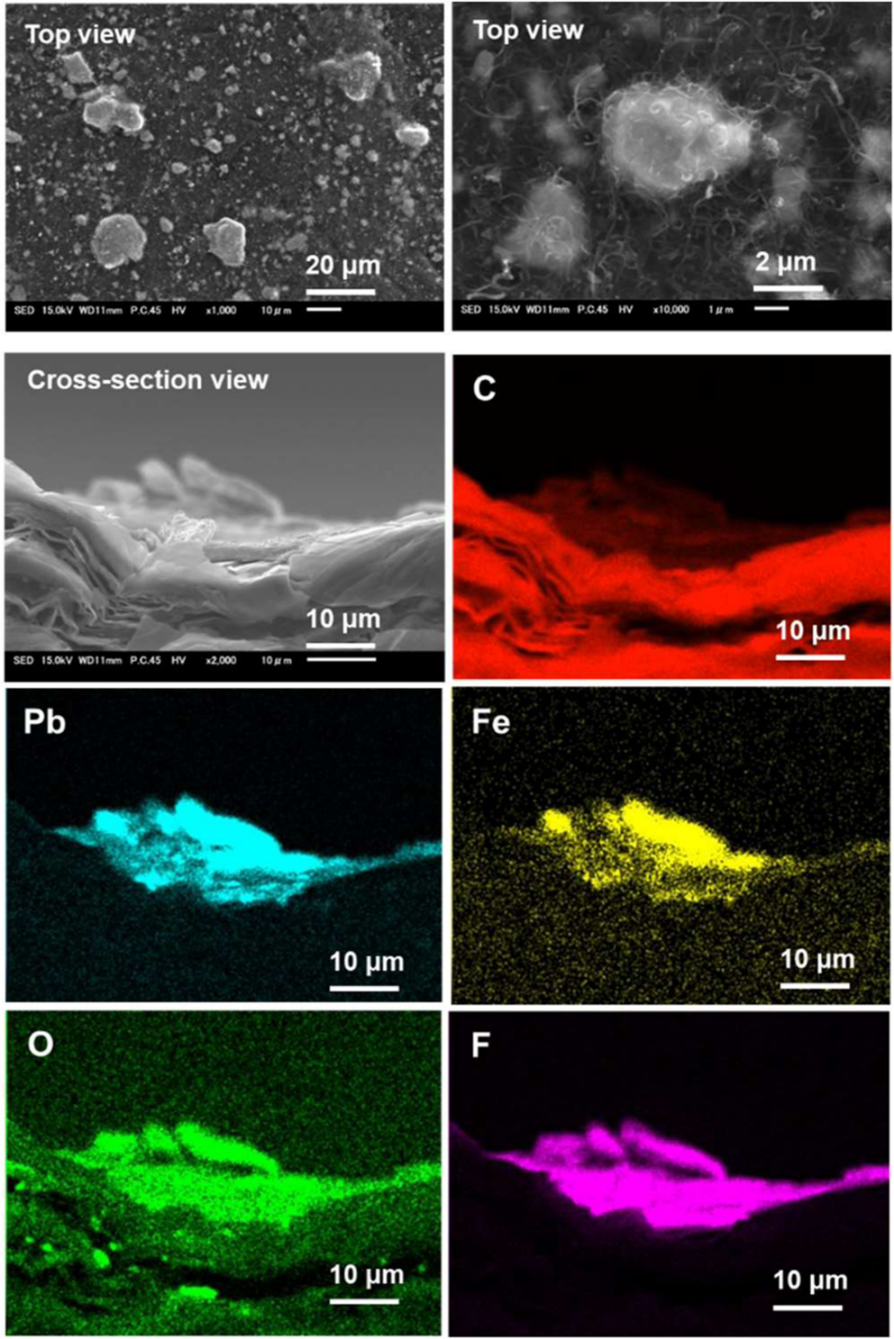
SEM–EDS analysis of the fabricated Pb_3_Fe_2_O_5_F_2_–CNT/GS electrode.

Electrodes were prepared by mixing a certain amount of CNT as a conductive support with the synthesized Pb_3_Fe_2_O_5_F_2_ powder in a water–alcohol solution containing Nafion.^52^ The resultant catalyst ink was drop-casted onto GS used as a carbon substrate. SEM observations of the resultant Pb_3_Fe_2_O_5_F_2_–CNT/GS electrode showed that the oxyfluoride particles were dispersed on the substrate (Figure 3, top view). The deposition of an entangled thread-like material onto the particles was caused by the Nafion dispersion, which was used to improve the adhesion of the Pb_3_Fe_2_O_5_F_2_ particles and CNT. The EDS mapping images of the electrode’s cross-sectional view (Figure 3) show that F was more uniformly detected on the electrode surface compared with Pb and Fe because of the presence of F in the mixed Nafion. The high concentrations of C detected in the areas where Pb and Fe were observed correspond to the graphite sheet in the electrode. On the contrary, regions where the concentration of C on the graphite sheet was lower indicate the presence of the CNT mixed with the Pb_3_Fe_2_O_5_F_2_ powder. In this study, an EDS quantification for the electrode was not performed because of the difficulty in distinguishing peaks of light elements in the low energy range, which led to the Fe peak being partially overlapped by the F peak in the spectrum.

**Figure 4:**
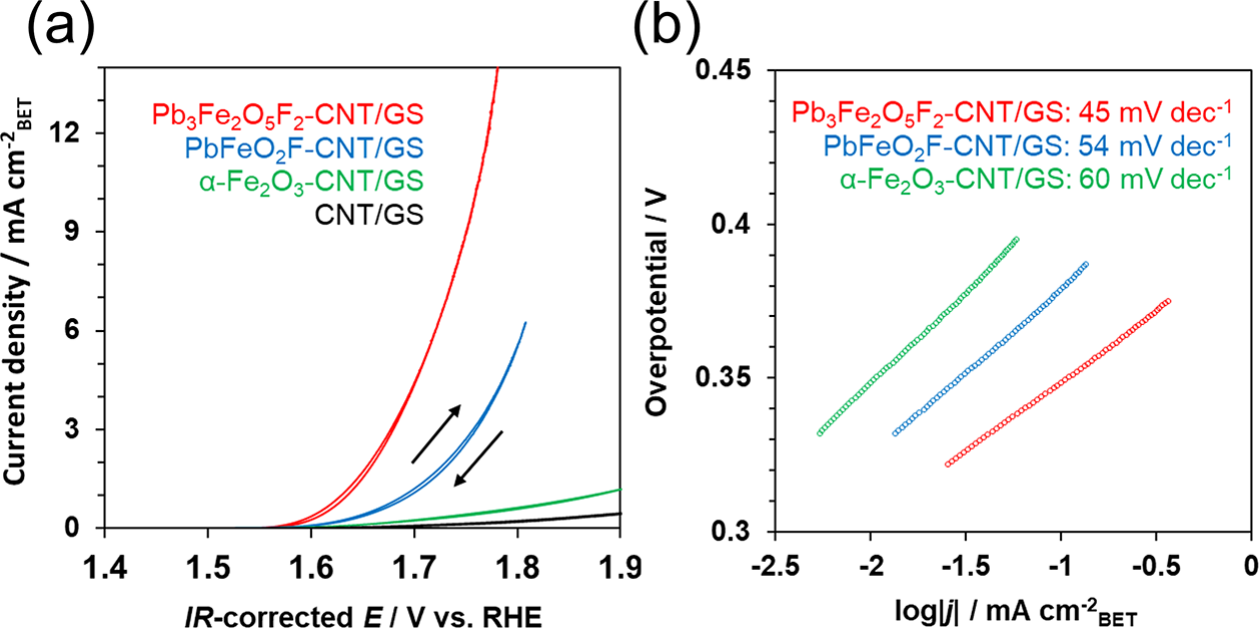
(a) Current–potential curves for Pb_3_Fe_2_O_5_F_2_–CNT/GS, PbFeO_2_F−CNT/GS, α-Fe_2_O_3_–CNT/GS, and CNT/GS electrodes in 1 M KOH (pH 14). (b) Tafel plots estimated from current–potential curves in the left figure. The respective electrode Tafel slopes (mV dec^–1^) are shown in the inset values. The amount of loading catalysts was 0.180 mg cm^–2^ on the electrodes.

PbFeO_2_F–CNT/GS, α-Fe_2_O_3_–CNT/GS, and CNT/GS electrodes were prepared by using a procedure similar to that used for the Pb_3_Fe_2_O_5_F_2_–CNT/GS electrode. The α-Fe_2_O_3_ sample was used as a raw material for the Pb_3_Fe_2_O_5_F_2_ synthesis. Electrochemical measurements were conducted for the as-prepared electrodes in an aqueous 1 M KOH solution (Figure 4a). The main CV scans were performed sequentially after a pretreatment of 20 CV scans in the same potential range. The current densities of the as-prepared electrodes were normalized with respect to the corresponding BET specific surface areas. To ensure an accurate comparison of the OER activities of the electrodes, it is necessary to normalize the current densities by the active surface area because the number of active (metal) sites in the water oxidation process is proportional to the active surface area on the electrocatalyst. Among the examined electrodes, the Pb_3_Fe_2_O_5_F_2_–CNT/GS electrode exhibited the steepest irreversible curve associated with water oxidation. The CNT/GS electrode showed a current density much lower than those of the oxyfluoride-deposited Pb_3_Fe_2_O_5_F_2_–CNT/GS and PbFeO_2_F−CNT/GS electrodes. Furthermore, the anodic current for the α-Fe_2_O_3_–CNT/GS electrode did not substantially increase compared with that for the oxyfluoride-deposited electrodes. These results indicate that Pb_3_Fe_2_O_5_F_2_ exhibited superior OER activity to PbFeO_2_F, which has a different crystal structure. The results also indicate that the high anodic current observed for the oxyfluoride electrodes did not originate from the residual Fe-containing precursor used in the synthesis or the CNT conductive support.

The onset potential for water oxidation (*E*_onset_) was determined as the potential at which the current density reached 0.05 mA cm_BET_^–2^ by reference to a previous report.^65^ The overpotential for water oxidation (η) was determined by subtracting the theoretical water oxidation potential (+1.23 V vs RHE) from *E*_onset_. The η values for Pb_3_Fe_2_O_5_F_2_, PbFeO_2_F, and α-Fe_2_O_3_ were 0.34, 0.36, and 0.39 V, respectively. Meanwhile, the Tafel slopes for the electrodes were calculated from the respective current–potential curves per BET specific surface areas (Figure 4b). The Tafel slopes of the inset values (mV dec^–1^) became larger in the order Pb_3_Fe_2_O_5_F_2_, PbFeO_2_F, and α-Fe_2_O_3_, showing a tendency similar to the η values. The lower overpotential and smaller Tafel slope for Pb_3_Fe_2_O_5_F_2_ than those for PbFeO_2_F and α-Fe_2_O_3_ indicate that the electrochemical properties of the former material are superior to those of the other electrode materials. Comparing the η and Tafel slope for Pb_3_Fe_2_O_5_F_2_ with those for previously reported Fe-based electrocatalysts (Table S1) reveals that Pb_3_Fe_2_O_5_F_2_ has a smaller η and a smaller Tafel slope. These results indicate that Pb_3_Fe_2_O_5_F_2_ exhibits better electrochemical activity toward the OER than simple perovskite oxides that contain only Fe as the active metal without another active metal such as Cu, Ni, or Co in the composition. EIS measurements also confirmed the superior electrochemical properties of Pb_3_Fe_2_O_5_F_2_ (Figure S3). The charge transfer resistance (*R*_ct_) for the Pb_3_Fe_2_O_5_F_2_, PbFeO_2_F, and α-Fe_2_O_3_ electrodes was obtained by fitting the resultant Nyquist plots and was found to be 16, 22, and 45 Ω, respectively. The smaller *R*_ct_ for Pb_3_Fe_2_O_5_F_2_ indicates better electrochemical properties with respect to the OER kinetics.

We measured the amount of O_2_ evolved when the Pb_3_Fe_2_O_5_F_2_–CNT/GS electrode was used for constant-potential electrolysis at +1.6 V vs RHE for 3 h (Figure S4). O_2_ gas was produced stably for the entire duration, with a Faradaic efficiency of 93%. This result confirms that the anodic current was derived primarily from the oxidation of water.

**Figure 5:**
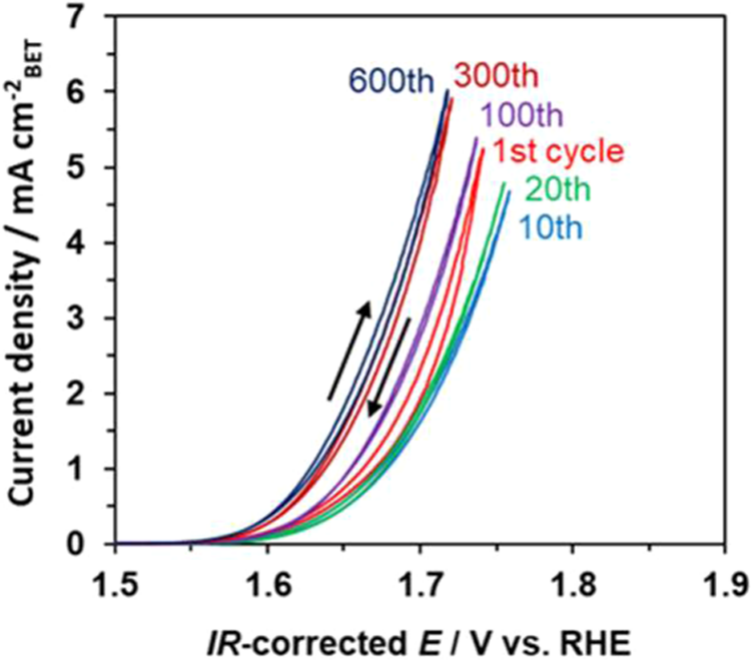
OER current–potential curves for the initial first cycle to the 600th cycle for the Pb_3_Fe_2_O_5_F_2_–CNT/GS electrode in 1 M KOH (pH 14).

Durability tests were conducted via CV for more than 600 cycles at a sweep rate of 10 mV s^–1^ (Figure 5). The anodic current decreased until the 10th cycle and then increased until the 300th cycle. The irreversible wave thereafter exhibited almost no change until the 600th cycle (corresponding to 18 h), indicating good durability of Pb_3_Fe_2_O_5_F_2_ during operation. The initial decrease in anodic current might be due to partial oxidative corrosion of the CNTs during the OER, as mentioned in a previous report.^66^ However, the subsequent increase might be attributable to the amorphization of the oxyfluoride surface during the OER, resulting in an increase in the electrochemically active surface area.^67,68^ For previously reported Fe-based composite oxides,^67,69,70^ the anodic current derived from the OER increased until a certain number of cycles during repeated CV scans for similar reasons. In particular, the amorphization was reported to be limited to a few nanometers on the surface when metal–oxygen bonds were more covalent than Fe–O bonds, preventing continuous dissolution and leading to improvements in both activity and stability.^70^ In the Pb_3_Fe_2_O_5_F_2_ case, Pb–O bonds should be more covalent than Fe–O bonds. Nevertheless, it should be emphasized that this amorphization effect is negligible when comparing the initial OER activities of different materials (Figure 4) and Pb_3_Fe_2_O_5_F_2_ prepared under different conditions, as discussed in the following section. This is because the electrochemical data for these comparisons were obtained after the 20 CV scans as the pretreatment, in which the promotional effect of amorphization on the OER activity is not prominent (Figure 5).

To examine the possibility of the decomposition of the oxyfluoride caused by the amorphization, in situ XAFS and SEM-EDS were conducted for the Pb_3_Fe_2_O_5_F_2_–CNT/GS electrode. The in situ XAFS measurement during controlled-potential electrolysis was performed for the Fe–K edge in the 1 M KOH aqueous solution (Figure S5). The obtained XANES spectra displayed almost no change in the electronic states of Fe in Pb_3_Fe_2_O_5_F_2_ from 0 h (before applying the potential) to 5 h. On the other hand, current density increased during the 5 h, consistent with the tendency of the durability test that was separately done (Figure 5). These results indicate that the bulk of the Pb_3_Fe_2_O_5_F_2_ particles on the electrode was stable during the OER. Besides, the SEM-EDS analysis was performed for the Pb_3_Fe_2_O_5_F_2_–CNT/GS electrodes after tests similar to those shown in Figure 5 in 300 and 600 cycles (Figure S6). It showed no clear difference between the samples after CV in 300 and 600 cycles, although the surface of the Pb_3_Fe_2_O_5_F_2_ particles became smooth compared to those before the test in Figure 3. The surface smoothing seems to be caused by partial amorphization on the Pb_3_Fe_2_O_5_F_2_ particles, as mentioned above. In addition, an ICP-OES analysis of the 1 M KOH electrolyte solution after the durability test revealed the dissolution of the Pb and Fe components (3.2 and 3.3 wt %, respectively) in the Pb_3_Fe_2_O_5_F_2_; this partial dissolution appears to be related to the amorphization that occurs only on the surface. Furthermore, the amounts of dissolved Pb, Fe, and F ions in the solution after the identical tests in Figure 5 and just immersion of the electrode for 18 h (not subject to an electrochemical measurement) were compared using the ICP-OES and a fluoride ion assay kit (Table S2). The dissolved amounts of the elements from the immersion for 18 h were smaller than those after the durability tests in 300 (9 h) and 600 (18 h) cycles. It means that the surface smoothing on the Pb_3_Fe_2_O_5_F_2_ particles was likely caused not by simple dissolution of the particles in the 1 M KOH solution but by the amorphization. On the basis of these results, sufficient structural stability of Pb_3_Fe_2_O_5_F_2_ was experimentally demonstrated with a negligible loss of fluorine content.

### Facet-Dependent Activity of the Pb_3_Fe_2_O_5_F_2_ Electrocatalyst

**Figure 6:**
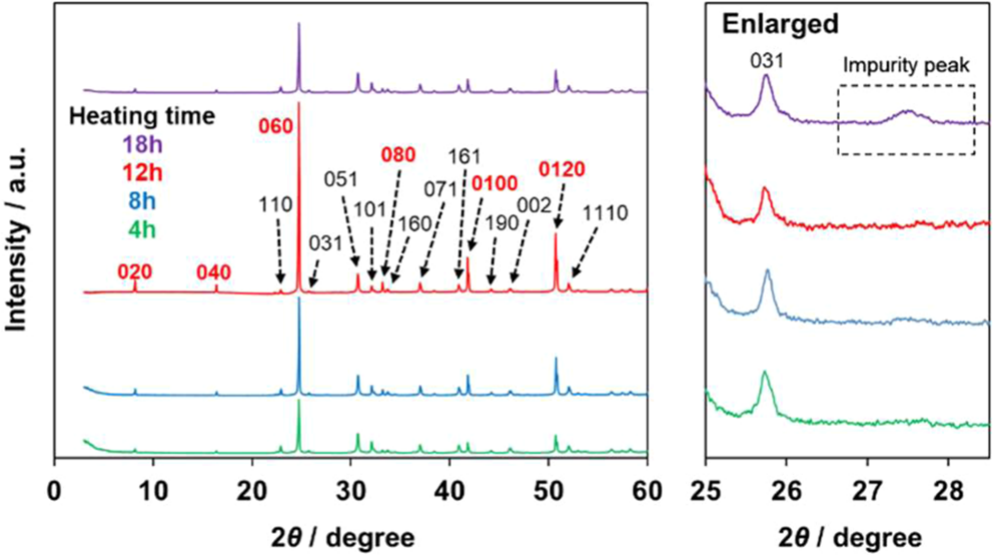
XRD patterns for Pb_3_Fe_2_O_5_F_2_ samples prepared with heating times of 4, 8, 12, and 18 h.

To investigate the reason for the higher OER activity of Pb_3_Fe_2_O_5_F_2_, we observed the XRD peak intensity for certain crystal facets of the Pb_3_Fe_2_O_5_F_2_ particles (Figure 6). In the synthesis of Pb_3_Fe_2_O_5_F_2_ by the solid-state reaction, the heating time was varied from 12 to 4, 8, and 18 h. XRD measurements were performed, and the resultant XRD patterns for the samples heated for 4, 8, or 12 h matched the peak positions of the reference data, confirming the synthesis of single-phase Pb_3_Fe_2_O_5_F_2_. At this time, the intensity of the XRD peaks corresponding to the (0*k*0) facets in the stacking (*b*-axis) direction of the layered structure increased with increasing heating time to 12 h (see the red-highlighted (0*k*0) facets in Figure 6). One of the (0*k*0) facets with Fe atoms, which are the active species for water oxidation,^71,72^ is the (060) facet with the highest peak intensity in the XRD patterns. In contrast, the peak intensity corresponding to the other facets showed no substantial change with increasing heating time (see the other black-lighted facets in Figure 6).

For a more detailed investigation of the above trend in the XRD patterns, Rietveld refinement was conducted using the diffraction data of the 4, 8, 12, and 18 h samples (Figure S7). Changes in the lattice parameters with respect to the heating time were almost negligible (see parameters of *a*, *b*, *c*, β, and *V* in Table S3), indicating that the obtained samples were homogeneous single phase. On the other hand, average crystallite sizes estimated from the refinement data increased from the 4 h to the 12 h sample, in which any effect from the equipment was compensated using an XRD pattern of Si powder. These results indicate that crystal growth occurred with the heating time increased to 12 h.

Besides, the significant increase in the intensities of 0*k*0 reflections is attributable to a sample orientation. The degree of the orientation was refined using March–Dollase function,^73^ and a decrease in the parameter *r* of the refinement data (see inset values in Figure S7) demonstrated that the 0*k*0 orientation was more pronounced as the heating time increased to 12 h. Crystallite sizes were estimated for three peaks, 020, 001, and 100, using the Scherrer equation (Table S4). The results displayed that the crystallite sizes along the *b*-axis exhibited minimal change with the varied heating time, but the crystallites in the *ac*-plane displayed crystal growth behavior as the increased heating time, which indicates that the Pb_3_Fe_2_O_5_F_2_ crystallites grew to spread in the *ac*-plane as the heating time was increased from 4 to 12 h. Therefore, the change in the degree of 0*k*0 orientation is presumed to signify a modification in the aspect ratio of the crystallites with specific crystal growth of the (0*k*0) facets.

Unlike the 4 and 8 h samples, the (0*k*0) peak intensities for the 18 h sample became lower than those of the 12 h sample. This result might be attributable to the heating time being too long, and the sample was likely to have unknown impurities as displayed by a broad peak observed in the range 27–28° (see the enlarged XRD patterns in Figure 6).

SEM observations of the Pb_3_Fe_2_O_5_F_2_ powder samples prepared with each heating time show stacking and aggregation of layered compounds but no substantial difference among the samples (Figure S8). Even higher magnification observation by field emission-SEM (FE-SEM) displayed no obvious difference in the particle size distribution and morphology between the 4 and 12 h samples of Pb_3_Fe_2_O_5_F_2_ (Figure S9). The lack of substantial changes in the particle size distribution and morphology is consistent with the tendency of the BET specific surface areas for the samples to range from only 1 to 2 m^2^ g^–1^ (see inset values in Figure S8). The little difference could thus be identified by SEM, but crystal growth in the *ac*-plane (i.e., perpendicular to the stacking direction) should occur judging from the integrated results of the above Rietveld refinement as well as the FE-SEM measurements. In fact, it has been reported in some layered perovskites that the reflection intensity in the stacking direction increased with increasing the synthesis temperature while maintaining in-plane reflection intensity and that a significant growth of the two-dimensional (2D) plane perpendicular to the stacking direction was seen by SEM observations.^74,75^

To examine the difference in the electronic (oxidation) states among the Pb_3_Fe_2_O_5_F_2_ powder samples, we analyzed the samples by XPS (Figure S10) and XAFS (Figure S11). In the XPS spectra, the binding energy values for all the relevant elements (Pb, Fe, O, and F) varied only slightly among the Pb_3_Fe_2_O_5_F_2_ samples where surface atomic ratios of the elements also showed only a slight change to be stoichiometric for Pb_3_Fe_2_O_5_F_2_ (Table S5). The electronic states for Fe in the Pb_3_Fe_2_O_5_F_2_ samples were examined by XAFS measurements. The X-ray absorption near edge structure (XANES) spectra show a steep rise of the absorption at ∼7120 eV, which is similar to that for α-Fe_2_O_3_, irrespective of the heating time during Pb_3_Fe_2_O_5_F_2_ synthesis (Figure S11a). Fe atoms in all of the Pb_3_Fe_2_O_5_F_2_ samples were found to be mainly trivalent cations. In addition, the absorption edge position and shape were almost unchanged among the Pb_3_Fe_2_O_5_F_2_ samples. Identical spectral shapes were also confirmed by extended X-ray absorption fine structure (EXAFS) analysis (Figure S11b). These XPS and XAFS results indicate that the unique crystal growth on the (0*k*0) facet affected neither the surface nor the bulk electronic states of the Pb_3_Fe_2_O_5_F_2_. Thus, the main difference among the Pb_3_Fe_2_O_5_F_2_ samples prepared with different heating times was only the specific crystal growth of the (0*k*0) facets.

**Figure 7:**
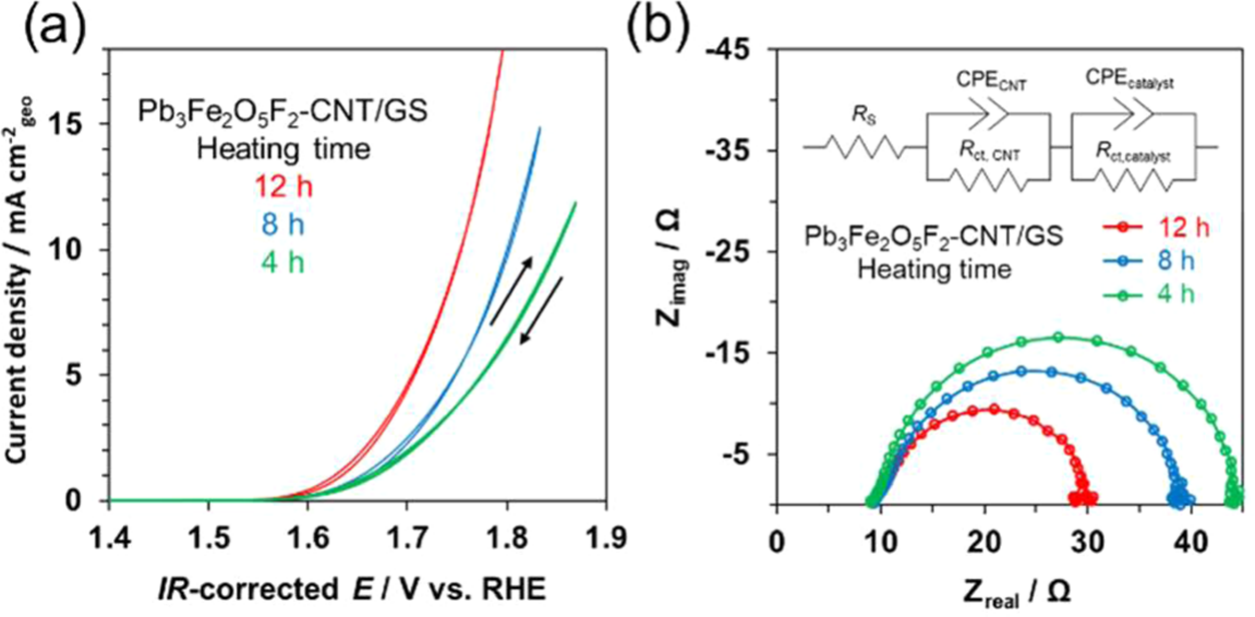
(a) Current–potential curves for Pb_3_Fe_2_O_5_F_2_–CNT/GS electrodes prepared using Pb_3_Fe_2_O_5_F_2_ samples synthesized with heating times of 4, 8, and 12 h. The electrodes were immersed in 1 M KOH (pH 14), and the current–potential curves were recorded at a sweep rate of 10 mV s^–1^. (b) Nyquist plots for Pb_3_Fe_2_O_5_F_2_–CNT/GS electrodes in the same solution at +1.7 V vs RHE, where frequency was varied from 100 kHz to 0.1 Hz. The catalyst loading on the electrodes was 0.108 mg cm^–2^.

Pb_3_Fe_2_O_5_F_2_–CNT/GS electrodes were prepared in the same manner as above by using the Pb_3_Fe_2_O_5_F_2_ samples heated for 4, 8, and 12 h during their synthesis. Electrochemical measurements were performed for the as-prepared electrodes (Figure 7a). The sample heated for 18 h was excluded from the measurements because of the presence of unknown impurities, as previously mentioned. The OER activities of the Pb_3_Fe_2_O_5_F_2_ electrodes increased with increasing heating time, which strongly suggests that the (0*k*0) facets of Pb_3_Fe_2_O_5_F_2_ are responsible for the enhanced OER activity. EIS measurements were conducted on the Pb_3_Fe_2_O_5_F_2_–CNT/GS electrode (Figure 7b). With increasing heating time, the radius of the arcs in the Nyquist plots decreased, corresponding to the decrease in the *R*_ct_ values obtained by fitting the data using an equivalent circuit (Figure 7b, inset diagram). The *R*_ct_ values were 18, 27, and 33 Ω for the samples heated for 12, 8, and 4 h, respectively. These results show that the Pb_3_Fe_2_O_5_F_2_ samples with stronger (0*k*0)-facet peak intensities exhibited kinetically superior electrochemical properties and higher OER activities. As previously mentioned, the size distribution and particle morphology and the bulk and surface electronic states did not substantially differ among the Pb_3_Fe_2_O_5_F_2_ samples synthesized at different heating times. Therefore, the results strongly suggest that the specific crystal growth of the (0*k*0) facets in the Pb_3_Fe_2_O_5_F_2_ samples enhanced the OER activity.

### DFT Calculations to Investigate the Origin of Facet-Dependent Activity

As previously mentioned, the higher OER activity of Pb_3_Fe_2_O_5_F_2_ appears to originate from the specific crystal growth of the (0*k*0) facets aligned in the stacking direction of the layered structure. However, whether this higher activity is related to an increase in the exposed area of the facets and/or to improved crystallinity accompanying facet growth is unclear. Therefore, we carried out DFT calculations for an OER process to clarify the origin of the facet-dependent activity on Pb_3_Fe_2_O_5_F_2_. Note that comparison of the OER process in different materials by DFT calculations is possible only for the initial state of the catalyst. We therefore examined whether there had been possible changes in the local structure around the Fe atom in Pb_3_Fe_2_O_5_F_2_ already during the catalyst preparation and/or prior to the electrochemical measurements (see Figure S12 for details). As the result, no significant change could be identified in the Fe–K edge XANES and EXAFS spectra.

**Figure 8:**
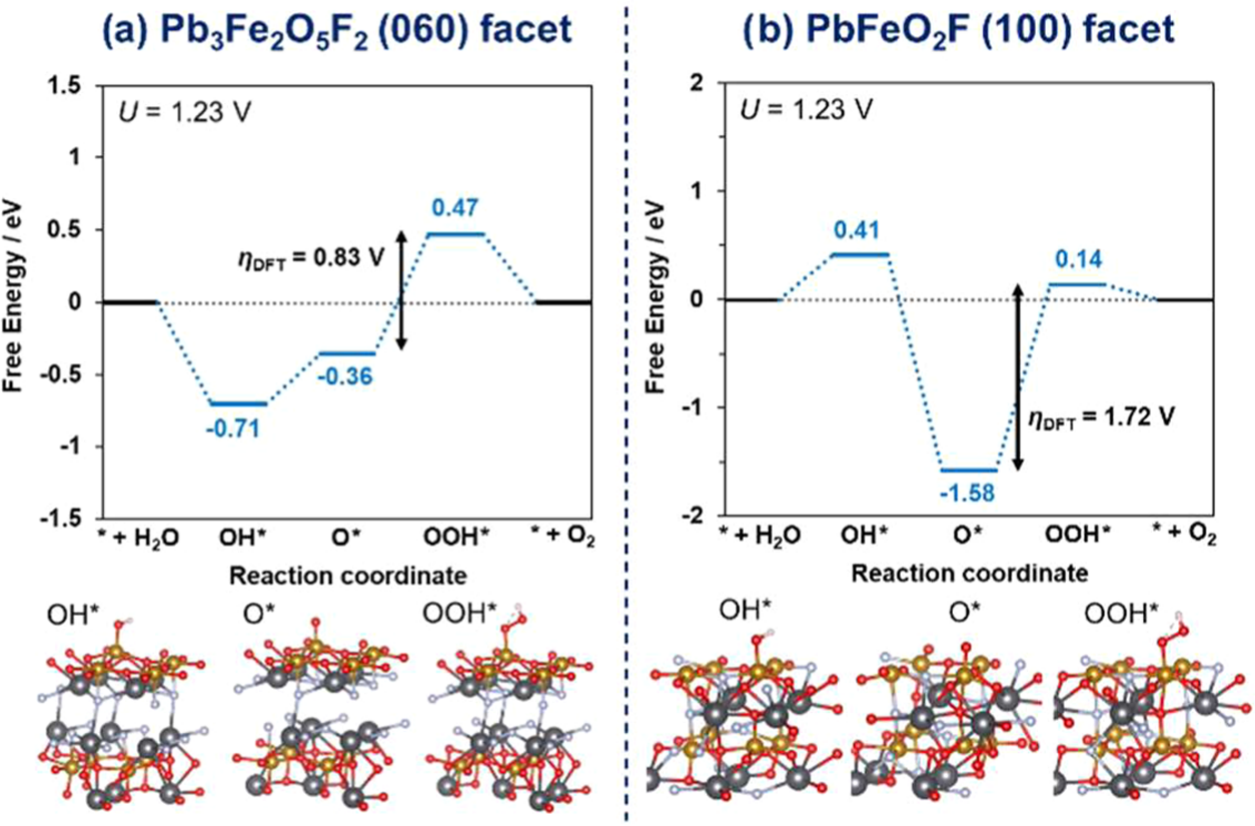
Gibbs energy diagrams for OER on (a) (060) facet of Pb_3_Fe_2_O_5_F_2_ and (b) (100) facet of PbFeO_2_F, as obtained by DFT calculations. The corresponding surface crystal structures for each state with adsorbed reaction intermediates are shown below (Pb: black, Fe: brown, O: red, F: white, H: beige). The change in the formation Gibbs free energy for each state is shown as the blue dotted line for the calculated values and as the black dotted line for the ideal values (when no overpotential is required). The diagrams show the energy states corresponding to the OER potential (+1.23 V vs RHE) applied toward the potential for hydrogen evolution reaction (0 V vs RHE) (voltage *U* = 1.23 V).

The theoretical overpotentials (η_DFT_) for water oxidation were estimated by DFT calculations and were compared between those of Pb_3_Fe_2_O_5_F_2_ and PbFeO_2_F (Figure 8). The reaction Gibbs free energy change Δ*G* for each elementary step for the OER process on each crystal facet of the two oxyfluorides was calculated. The calculations were performed by cutting the oxyfluoride structures at certain crystal planes. The η_DFT_ values were determined from the results because they correspond to the maximum Δ*G* values in the four elementary steps of the AEM and LOM processes. The metal cations that serve as active sites (Fe in this study) are sufficiently far from each other in perovskite structures, including layered perovskite materials, where water oxidation mainly proceeds via this AEM.^58,59,64,76^ In contrast, some perovskite oxides allowed to change AEM to LOM when an electronic state of active metal is changed by another metal substitution.^61,62,77^ However, the η_DFT_ values for the AEM process were much lower than those for the LOM process (Figure S13). Therefore, the AEM process was mainly considered in this work. The Experimental Section provides further details of the calculations.

Among the (0*k*0) facets of the Pb_3_Fe_2_O_5_F_2_, the (060) facet, which shows the highest XRD peak intensity in Figure 6, had Fe atoms on the plane; these Fe atoms function as active sites for water oxidation in Fe-based electrocatalysts.^71,72^ At first, the formation Gibbs free energy of the (060) facet with the Fe atoms on the surface was compared to those of (010), (020), and (040) facets with Pb atoms by DFT calculations. The (060) facet with the Fe atoms showed higher Gibbs energy than the (010), (020), and (040) facets with the Pb atoms, and the (060) facet was less stable than the (010), (020), and (040) facets (Table S6). However, the η_DFT_ value of the (060) facet (0.83 V) was clearly lower than that of the (010) facet (1.58 V) when calculated by DFT calculations for the AEM process (Figures 8a and S14). From these results, we focused on the facets with the Fe atoms as an electrochemically active surface to calculate theoretical overpotentials hereafter. Thus, the η_DFT_ value of the (060) facet was compared to that of the (001) facet in the layer direction (*ac*-plane) with exposed Fe atoms on the surface as well (Figure S15). The results show a lower η_DFT_ value for the (060) facet (0.83 V) than that for the (001) facet (1.38 V).

On the other hand, the η_DFT_ values for each facet of PbFeO_2_F, which were determined in the same manner as described above (Figure S16), were 1.72, 16.78, and 2.04 V for the (100), (110), and (111) facets, respectively. These facets correspond to the three peaks with the highest intensity in the XRD pattern for PbFeO_2_F to exclude equivalent facets such as (010) (Figure S1). The (100) facet displayed the lowest η_DFT_ values in the main facets. Besides, the (110) facet showed an abnormally high overpotential of 16.78 V, which indicates that OER does not proceed on the (110) facet in PbFeO_2_F. This high overpotential can be caused by the adsorption of reaction intermediates (OH, O, and OOH) not only on a Fe atom but also on a Pb atom adjacent to Fe in the (110) facet (see the surface crystal structures in Figure S16).

The η_DFT_ value for the (060) facet of Pb_3_Fe_2_O_5_F_2_ was 0.83 V, which is much lower than that for the (100) facet of PbFeO_2_F (1.72 V, Figure 8b). This calculated difference between the two η_DFT_ values demonstrated that Pb_3_Fe_2_O_5_F_2_ was clearly favorable for the OER compared to PbFeO_2_F because electrochemical reactions proceed with reaction pathways on the catalyst surface with the lowest overpotential. The difference of η_DFT_ was much larger than that of the experimentally obtained η values (0.02 V); however, DFT calculations typically overestimate theoretical overpotentials compared with their experimental values.^64^ In addition, an experimental η value depends on the method of estimation (here, the values at 0.05 mA cm_BET_^–2^). As a result, the exposure of the (060) facet on the Pb_3_Fe_2_O_5_F_2_ surface was important for the lower overpotential and higher OER activity of Pb_3_Fe_2_O_5_F_2_. It implies that the improved crystallinity accompanied by the facet growth did not mainly lead to the higher activity; rather, the increase in the area of the (0*k*0) facets affected it, given the improvement of the OER activity as a result of surface amorphization (Figure 5).

To explain the smaller theoretical overpotential on the (060) facet of Pb_3_Fe_2_O_5_F_2_, we focused on the number of electrons in the e_g_ orbitals of an active metal cation on the catalyst surface as a steady-state parameter to describe the OER activity. To maximize the OER activity, according to Sabatier’s principle, it is important to have a moderately strong bond between an active metal and an adsorbed intermediate species.^78^ The number of the e_g_ electrons of the active metal is an indicator of a moderately strong bond as a steady-state parameter, which is correlated with the activity in the perovskite oxides.^79^ The occupancy of electrons in the e_g_ orbitals is related to the strength of the σ-bond between a transition-metal cation and adsorbed reaction intermediates,^80^ where lower occupancy indicates a stronger bond and higher occupancy indicates a weaker bond. Indeed, OER activities in perovskite and spinel oxides have been empirically found to be maximal when the e_g_ electron number is close to 1.2.^79,81^ In addition, the e_g_ electron numbers are 1 and 2 for Fe^4+^ and Fe^3+^, respectively, if Fe has a high-spin electron configuration. Applying this measure, when the charge of Fe is closer to +4, the electronic state is favorable for enhanced OER activity. In fact, the Fe charge calculated from DFT calculations tends to be −1 less than the actual Fe charge from composition.^82^ Therefore, we add a charge of +1 to the calculated charge to compare with the +4 charge.

**Figure 9:**
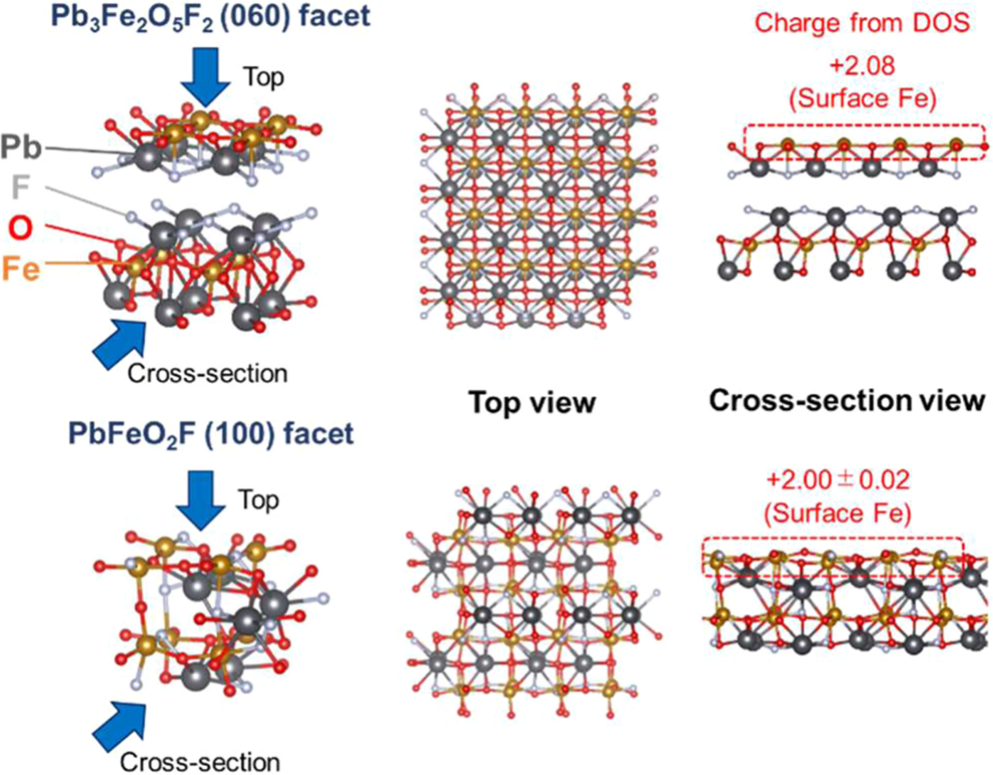
Top and cross-sectional views of crystal structures cut out in Pb_3_Fe_2_O_5_F_2_ (060) and PbFeO_2_F (100) facets and optimized by DFT calculations (Pb: black, Fe: brown, O: red, F: white). The charges of the Fe on each site calculated from the density of states are shown in the cross-sectional views. ±0.02 in the Fe charge of the PbFeO_2_F cross-sectional view represents the standard deviation for Fe charges on the surface.

We used DFT calculations of the surface crystal structures to obtain the charge of Fe as the active site from the density of states (DOS). Using the DOS, we determined the number of electrons in the outermost shell of the Fe atoms on the (060) facet and then used the number of electrons to calculate the charge of the Fe cations (Figure 9). For comparison, we also calculated the charge of those on the (100) facet of PbFeO_2_F in the same manner. The charge of Fe on the (060) facet of Pb_3_Fe_2_O_5_F_2_ was +2.08, which is higher than the charge of +2.00 on the (100) facet of PbFeO_2_F. Previous reports have indicated that even though the difference in charge values obtained from DFT calculations differs by two decimal places, it makes a significant difference in the actual OER activity.^83−85^

The layered structure of Pb_3_Fe_2_O_5_F_2_ separated the surface bilayer from the underlying bulk layer (see the cross-sectional view in Figure 9). This geometry likely caused F^–^ ions interacting locally with the Fe on the (060) facet to decrease the electron density and increase the positive charge on the Fe via their strong electron-withdrawing behavior. By contrast, the surface bilayer of PbFeO_2_F was continuous with the underlying bulk layer because of the cubic perovskite structure, resulting in less interaction locally with the Fe on the (100) facet. The more positive charge of +2.08 on the (060) facet of Pb_3_Fe_2_O_5_F_2_ than that of PbFeO_2_F is attributable to the localized interaction of F^–^ ions with the surface Fe. In addition, the localization effect appears not to be directly associated with bulk properties (e.g., the electronic band structure) in Pb_3_Fe_2_O_5_F_2_. Consequently, the level of the OER activity of Pb_3_Fe_2_O_5_F_2_ did not decrease after the amorphization on the surface (Figure 5).

## Conclusions

A layered perovskite oxyfluoride, Pb_3_Fe_2_O_5_F_2_, was used to prepare a Pb_3_Fe_2_O_5_F_2_–CNT/GS electrode with CNT and GS as conductive carbon support and a carbon substrate, respectively. The Pb_3_Fe_2_O_5_F_2_ electrode showed higher OER activity than a PbFeO_2_F electrode prepared in the same manner. In addition, an OER durability test for the Pb_3_Fe_2_O_5_F_2_ electrode demonstrated high stability for longer than 18 h, with a small degree of improvement in the OER activity by amorphization of the surface. An electrochemical investigation using Pb_3_Fe_2_O_5_F_2_ samples synthesized with different heating times revealed (0*k*0) facet-preferred OER activity. In addition, DFT calculations for the OER process to compare different facets of Pb_3_Fe_2_O_5_F_2_ and PbFeO_2_F revealed that the Pb_3_Fe_2_O_5_F_2_ (060) facet with Fe sites had a lower theoretical OER overpotential than that of the PbFeO_2_F (100) facet. This was attributed to a moderately strong bond between the active Fe sites and reaction intermediates as a result of the formed local interaction of F^–^ ions with the Fe sites in the layered structure.

Although crystal-facet-dependent OER activity has been reported in some metal oxide OER catalysts, little is known of such activity in mixed-anion compounds. The results of the present work clearly provide new insights into OER catalysts with heteroanions such as F^–^ ions from the viewpoint of crystal facets and the local coordination environment; these catalysts do not rely on precious/rare metals. We also note that further improvements in the OER activity of Pb_3_Fe_2_O_5_F_2_ are expected as a result of reducing the particle size and more defined facet control by using an appropriate synthetic method. Research along this line is underway in our laboratory.

## Supporting Information Available

Additional characterization; electrochemical and DFT calculation data; and comparison with the previously reported iron-based electrocatalysts (PDF)
