# Supplementary material for: Iron-Based Layered Perovskite Oxyfluoride Electrocatalyst for Oxygen Evolution: Insights from Crystal Facets with Heteroanionic Coordination
Source: J Am Chem Soc. 2024 Nov 13;146(47):32343–55. doi: 10.1021/jacs.4c05740 (PMC11613499; doi:10.1021/jacs.4c05740)
Supplement: Supplementary file 1 — ja4c05740_si_001.pdf [file ja4c05740_si_001.pdf]

Supporting Information (SI)

Iron-Based Layered Perovskite Oxyfluoride Electrocatalyst for Oxygen Evolution: Insights from Crystal Facets with Heteroanionic Coordination

*Ryusuke Mizuochi,[1] Yuuki Sugawara,[2] Kengo Oka,[3] Yoshiyuki Inaguma,[4] Shunsuke Nozawa,[5] Toshiyuki Yokoi,[2] Takeo Yamaguchi,[2] and Kazuhiko Maeda[1,6]**

[1] Department of Chemistry, School of Science, Institute of Science Tokyo, 2-12-1-NE-2 Ookayama, Meguro-ku, Tokyo 152-8550, Japan.

[2] Institute of Integrated Research, Institute of Science Tokyo, 4259 Nagatsuta-cho, Midori-ku, Yokohama, Kanagawa 226-8501, Japan.

[3] Department of Applied Chemistry, Faculty of Science and Engineering, Kindai University, 3-4-1 Kowakae, Higashi-osaka City, Osaka 577-8502, Japan.

[4] Department of Chemistry, Faculty of Science, Gakushuin University, 1-5-1 Mejiro, Toshima-ku, Tokyo 171-8588, Japan

[5] Institute of Materials Structure Science, High Energy Accelerator Research Organization, Tsukuba, Ibaraki 305-0801, Japan

[6] Research Center for Autonomous Systems Materialogy (ASMat), Institute of Science Tokyo, 4259 Nagatsuta-cho, Midori-ku, Yokohama, Kanagawa 226-8501, Japan.

*To whom correspondence should be addressed; Email: maeda@chem.sci.isct.ac.jp

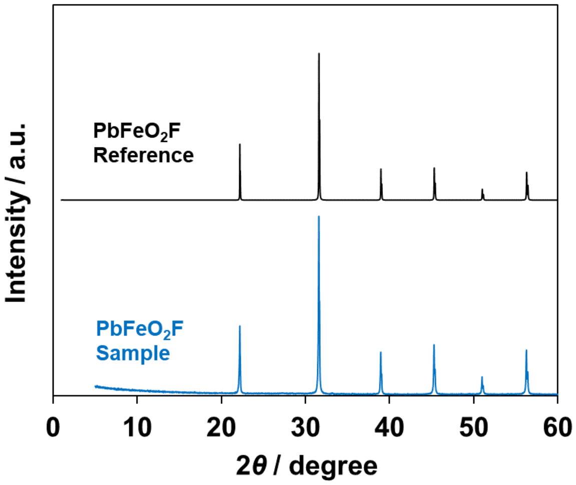

Figure S1. XRD patterns for synthesized $PbFeO_2F$ powder and its reference data (Ref. S1).

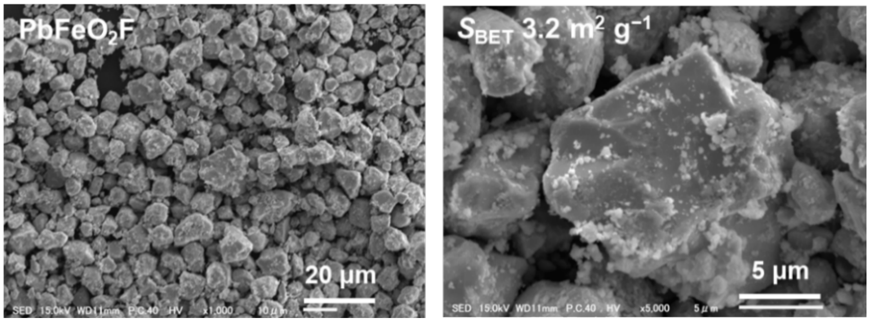

Figure S2. SEM images of synthesized $PbFeO_2F$. The inset value (S_{BET}, $m^2\ g^{-1}$) in the right image shows the BET specific surface area.

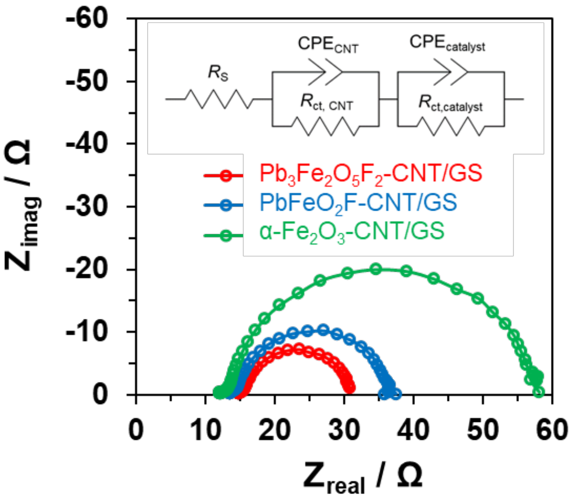

Figure S3. Nyquist plots for $Pb_3Fe_2O_5F_2$−CNT/GS, $PbFeO_2F$−CNT/GS, and *α*-Fe_2O_3−CNT/GS electrodes in 1 M KOH (pH 14) at +1.7 V vs. RHE. The frequency was varied from 100 kHz to 0.1 Hz.

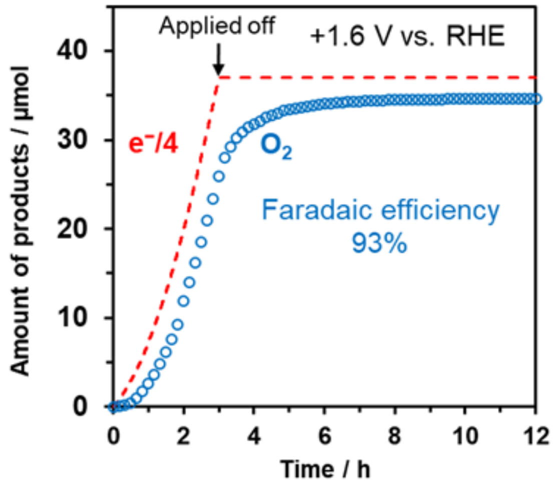

Figure S4. Quantification of O_2 evolved over $Pb_3Fe_2O_5F_2$–CNT/GS electrode in 1 M KOH (pH 14) at +1.6 V vs. RHE for 3 h. The Faradaic efficiency obtained from this measurement is shown.

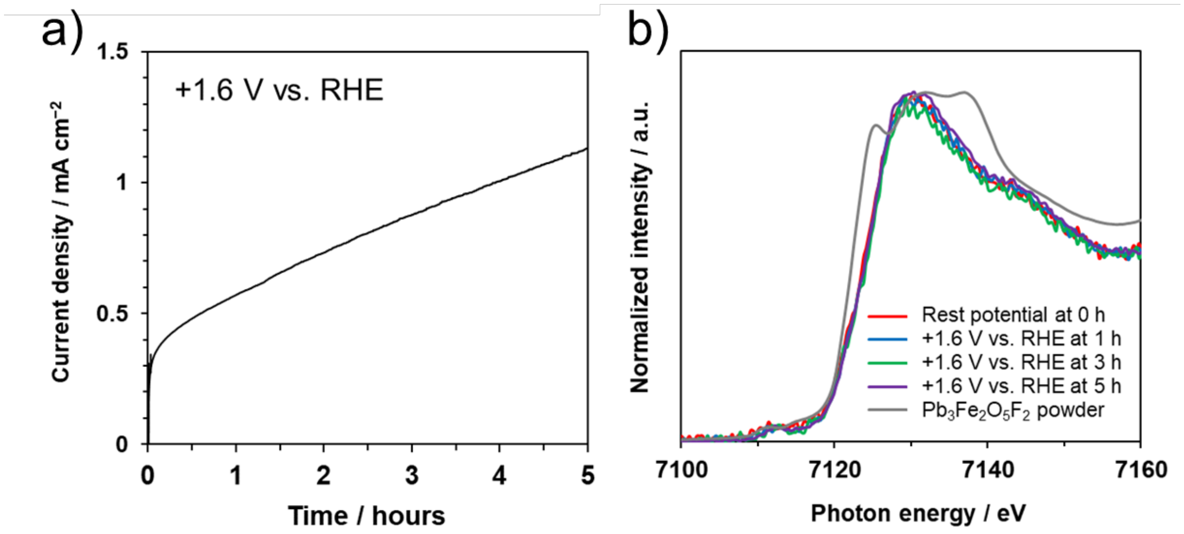

Figure S5. In-situ XAFS results of a) a current-time curve for a $Pb_3Fe_2O_5F_2$–CNT/GS electrode at +1.6 V vs. RHE in 1 M KOH (pH 14), and b) the corresponding Fe-K edge normalized XANES spectra acquired at 1, 3 and 5 h. XANES data for "0 h" before applying the potential and the $Pb_3Fe_2O_5F_2$ powder are shown for references. It is noted that the XANES spectra of the $Pb_3Fe_2O_5F_2$ electrodes were somewhat different from that of the corresponding $Pb_3Fe_2O_5F_2$ powder sample. This is attributable to much lower concentration of the $Pb_3Fe_2O_5F_2$ powder on the electrode than the densely packed powder sample. Similar spectral microstructure changes in samples with low concentrations of the target elements (or nano-sized samples) have been observed previously (Refs S2 and S3).

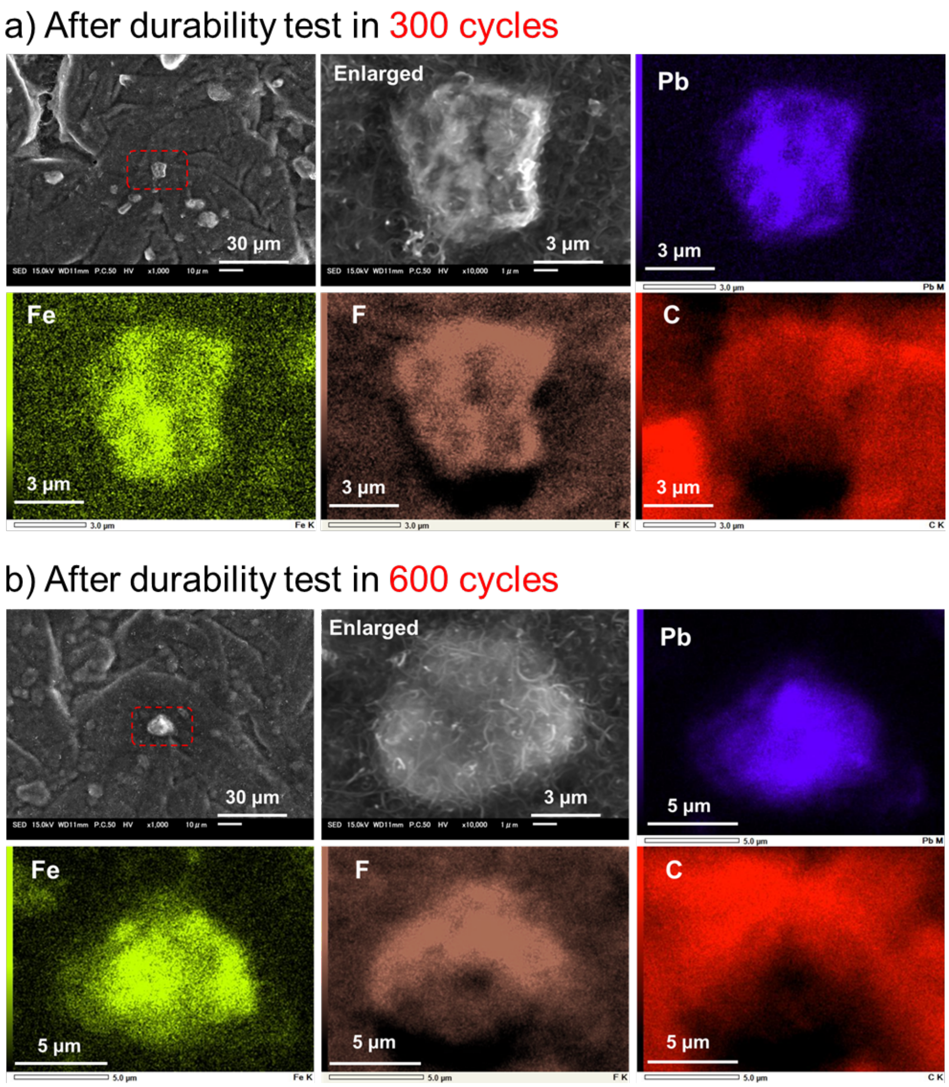

Figure S6. SEM−EDS analysis of $Pb_3Fe_2O_5F_2$−CNT/GS electrodes after durability tests of the CV similar to Figure 5 in the main text in (a) 300 and (b) 600 cycles.

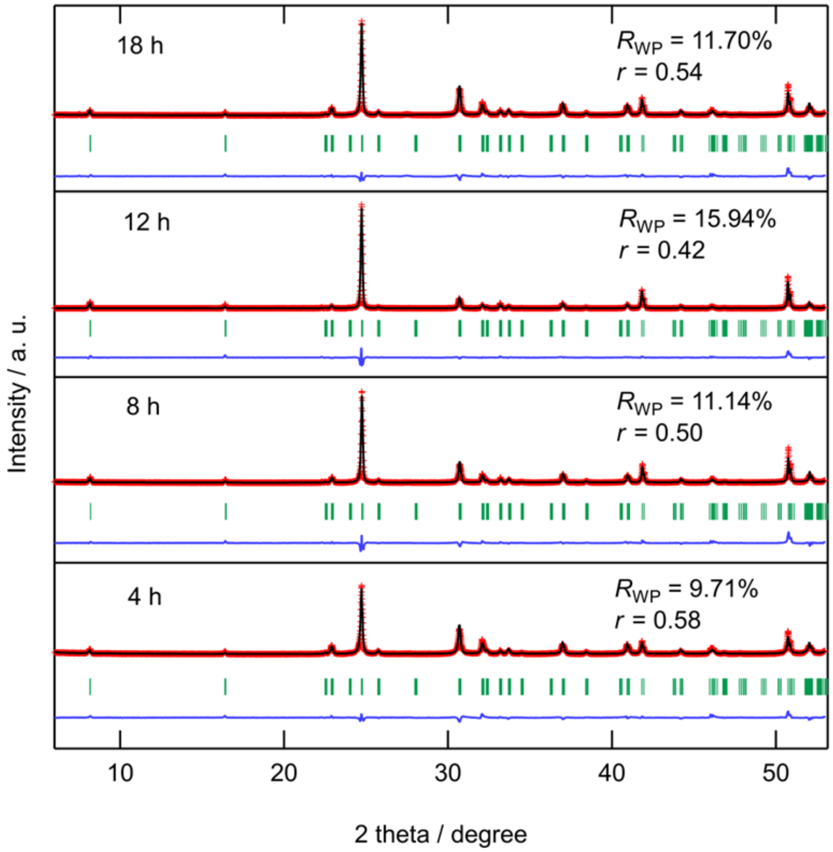

Figure S7. The results of Rietveld refinement performed for the XRD patterns of the $Pb_3Fe_2O_5F_2$ samples with varied annealing periods (4, 8, 12, and 18 h). Red crosses, solid black lines, and solid blue lines represent the observed, calculated, and difference intensities, respectively. The green ticks indicate the Bragg peak positions. The preferred 0*k*0 orientation was refined using the March-Dollase function (Ref. S4). The annotations indicate the reliability factors, R_{WP} and r parameters for the March-Dollase function. The reduction in r from unity indicates increase in the degree of 0*k*0 orientation.

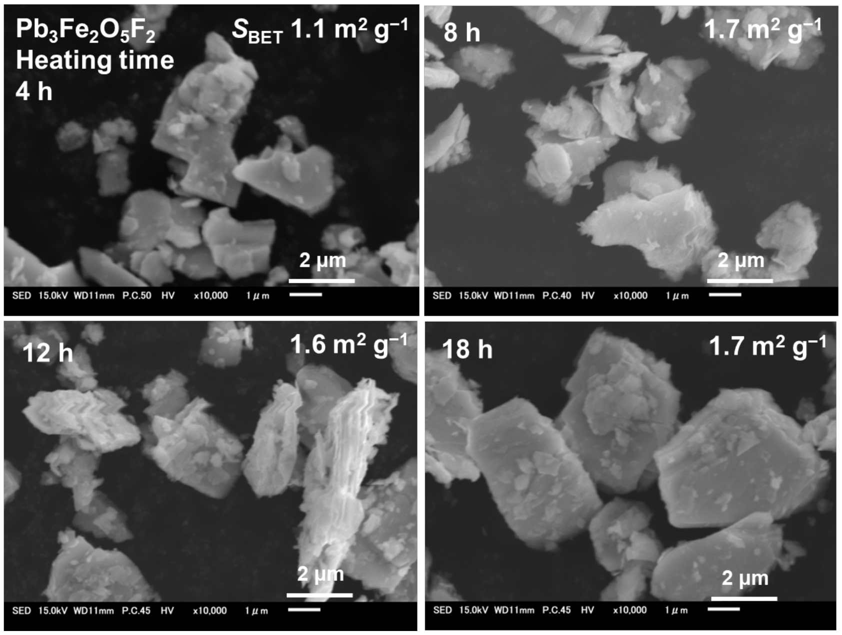

Figure S8. SEM images of $Pb_3Fe_2O_5F_2$ samples prepared with heating times of 4, 8, 12, and 18 h.

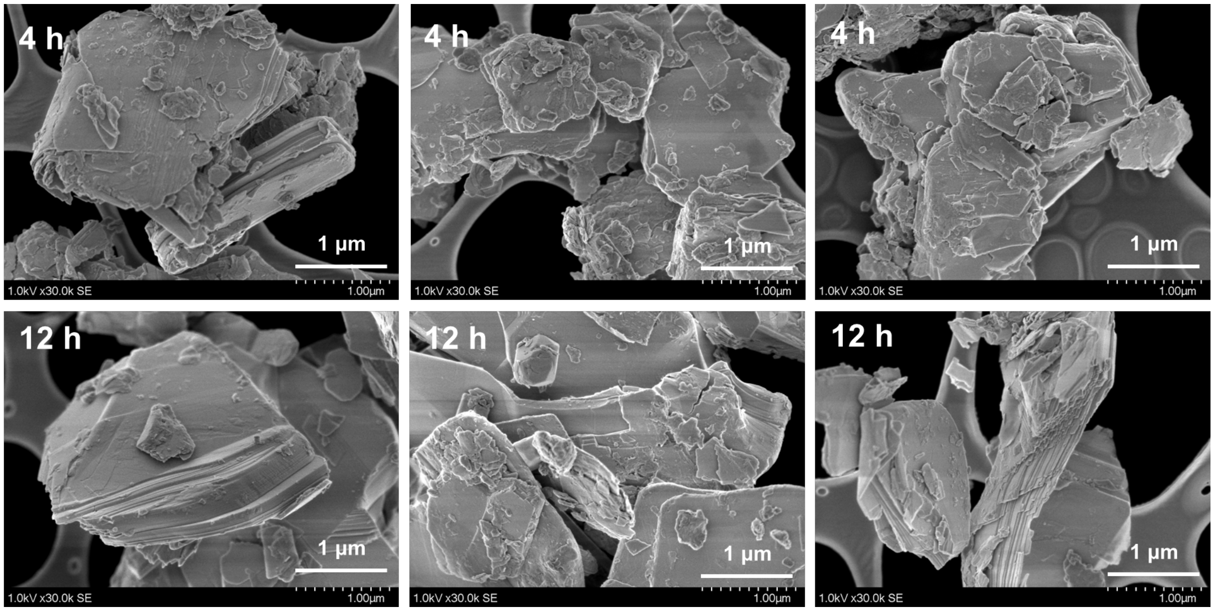

Figure S9. FE-SEM images of $Pb_3Fe_2O_5F_2$ samples prepared with heating times of (top) 4 h and (bottom) 12 h.

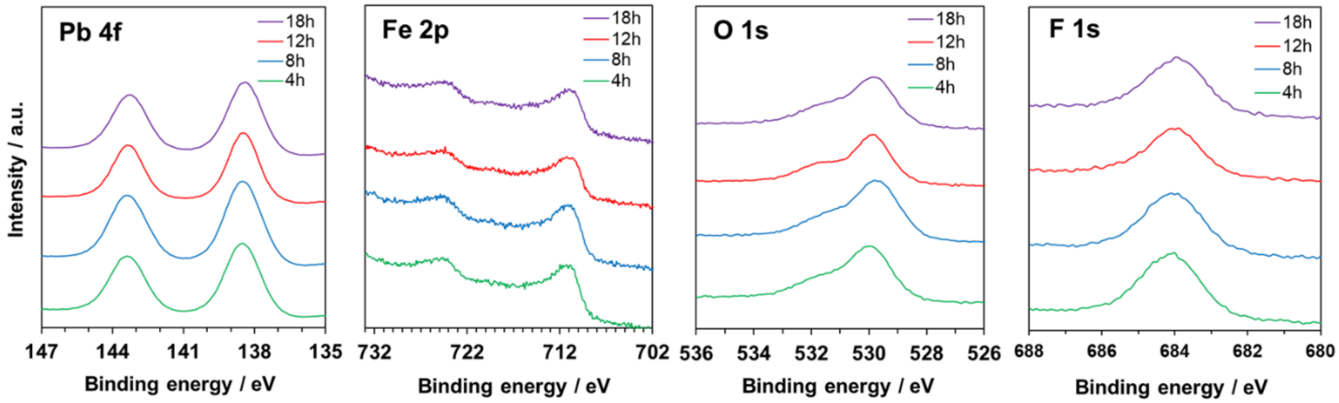

Figure S10. XPS spectra of Pb 4f, Fe 2p, O 1s, and F 1s of $Pb_3Fe_2O_5F_2$ samples prepared using different heating times during synthesis (4, 8, 12, and 18 h). The surface atomic ratios, as obtained from the XPS measurements, showed little variation among the samples; however, the stoichiometric ratios did not perfectly match those of $Pb_3Fe_2O_5F_2$ because of the presence of anionic (mainly F^-) defects (Table S5).

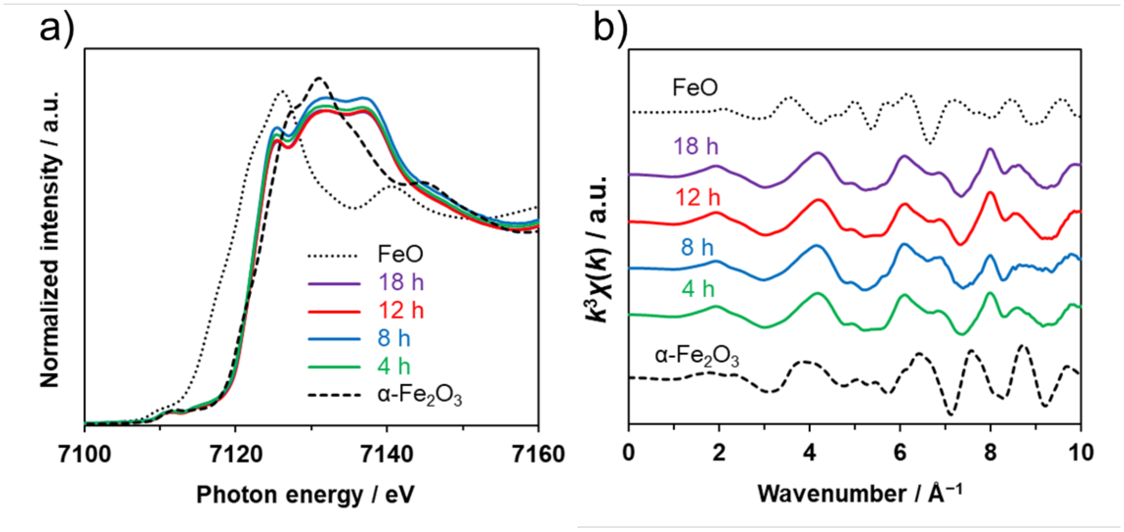

Figure S11. Fe-K edge a) normalized XANES and b) k^3-weighted EXAFS spectra of $Pb_3Fe_2O_5F_2$ samples prepared using different heating times during synthesis (4, 8, 12, and 18 h). Spectra of FeO and α-Fe_2O_3 are included for reference.

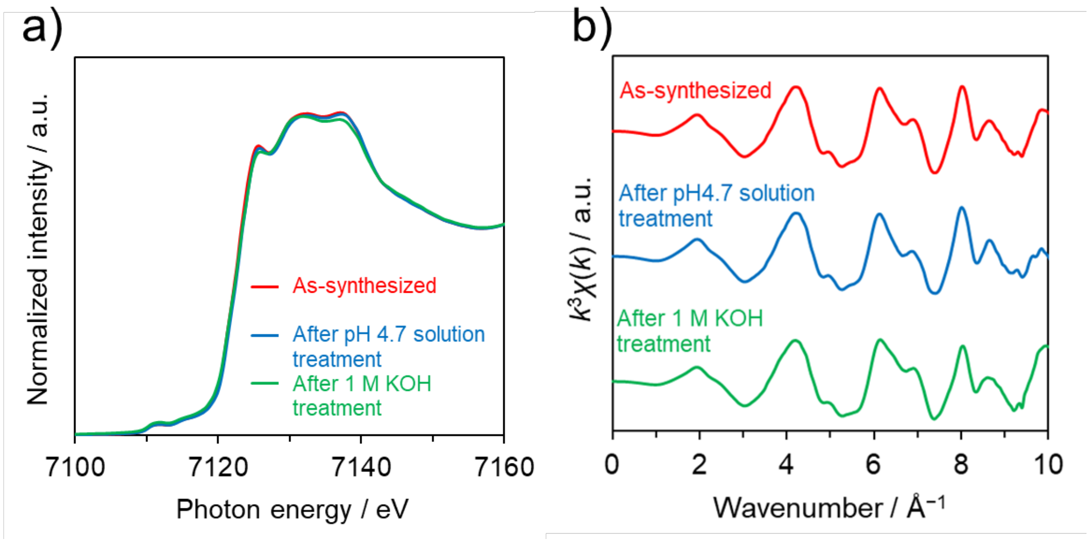

Figure S12. Fe-K edge a) normalized XANES and b) k^3-weighted EXAFS spectra of $Pb_3Fe_2O_5F_2$ of 12 h samples subject to two treatments. The detail of the treatments is described below.

Preparation of the Investigated Samples. Initially, we attempted to conduct characterization of $Pb_3Fe_2O_5F_2$ after isolation from the catalyst ink containing isopropanol, water, Nafion and MWCNT. However, it was not possible to do it because Nafion and MWCNT hindered the isolation. Thus, a chemically equivalent catalyst ink was prepared as follows, assuming that MWCNT does not give any chemical stimuli to $Pb_3Fe_2O_5F_2$ due to its chemical inertness and that Nafion releases protons from the sulfonic acid ($–SO_3H$) groups.

0.1 M H_2SO_4 was added to an aqueous isopropanol solution (50 vol.%) to adjust pH 4.7 while stirring. Then, an aliquot of 33 mL was moved to a glass vial containing 100 mg of $Pb_3Fe_2O_5F_2$, and sonicated for 2 h. After the sonication, the suspension was filtered to collect the solid sample, followed by drying in an oven at 343 K overnight. The resulting solid (70 mg) was then subject to immersion in 1 M KOH aqueous solution (30 mL) for 30 min. The solid was collected in the same manner. The treated $Pb_3Fe_2O_5F_2$ samples were examined by XAFS, as shown above.

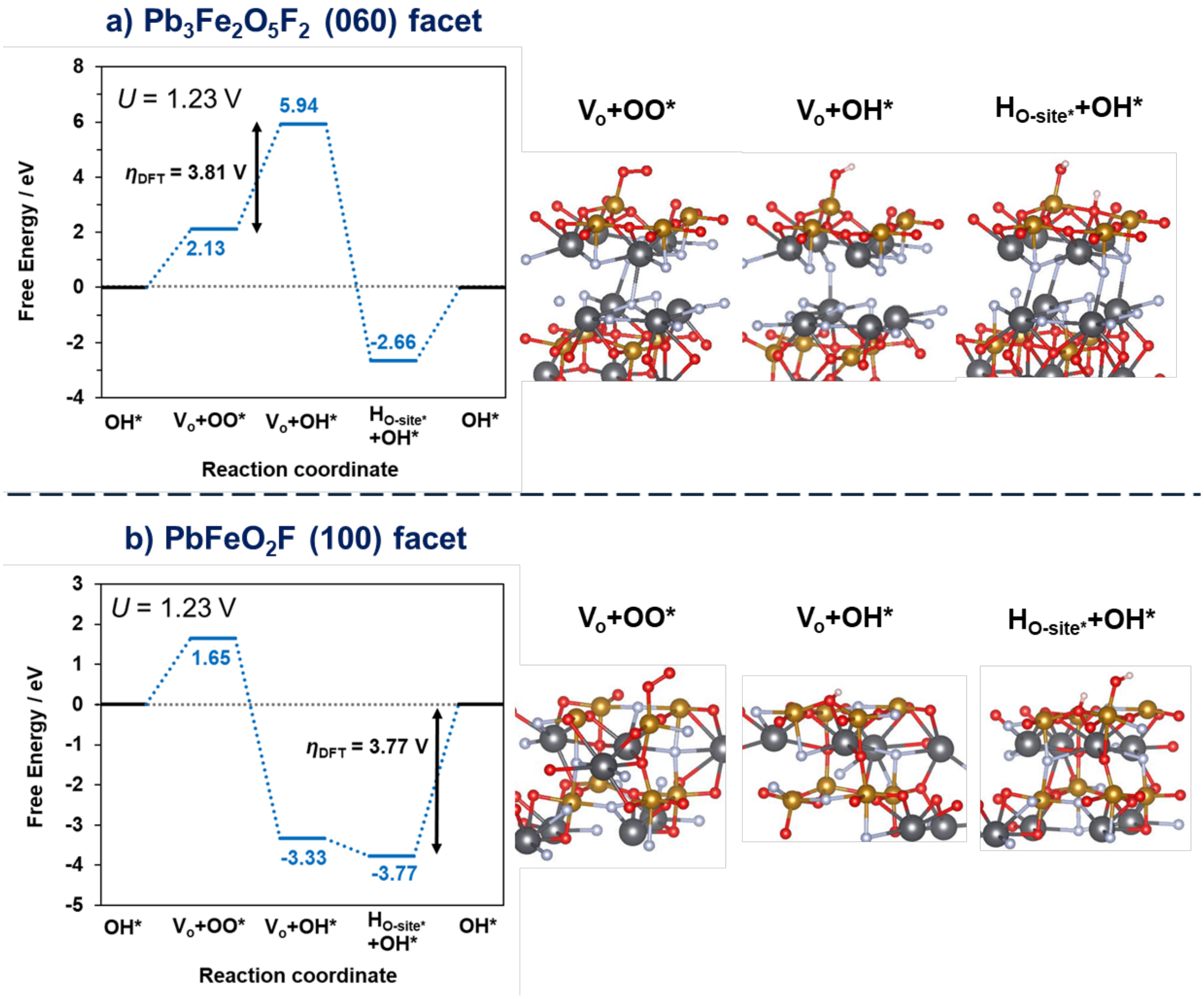

Figure S13. Gibbs energy diagrams for OER on a) (060) facet of $Pb_3Fe_2O_5F_2$ and b) (100) facets of $PbFeO_2F$, as obtained by DFT calculations through the LOM process. The corresponding surface crystal structures for each state with adsorbed reaction intermediates are shown below (Pb: black, Fe: brown, O: red, F: white, H: beige). The change in the formation Gibbs free energy for each state is shown as the blue dotted line for the calculated values and the black dotted line for the ideal values (when no overpotential is required). All the diagrams show the energy states corresponding to the OER potential (+1.23 V vs. RHE) applied toward the potential for hydrogen evolution reaction (0 V vs. RHE) (voltage U = 1.23 V). The LOM process with each elementary process was referred to Ref. S5.

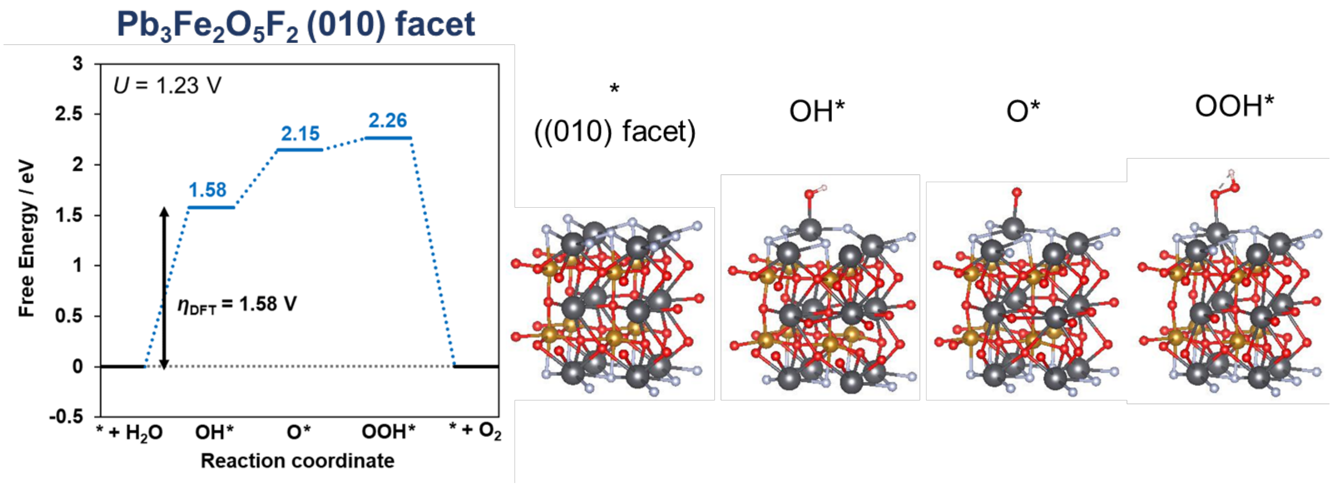

Figure S14. A Gibbs energy diagram for OER on a (010) facet of $Pb_3Fe_2O_5F_2$, as obtained by DFT calculations through the AEM process, and the corresponding surface crystal structure for each state with adsorbed reaction intermediates (Pb: black, Fe: brown, O: red, F: white, H: beige). Notations and conditions except for mentioned above are the same as in Figure S13.

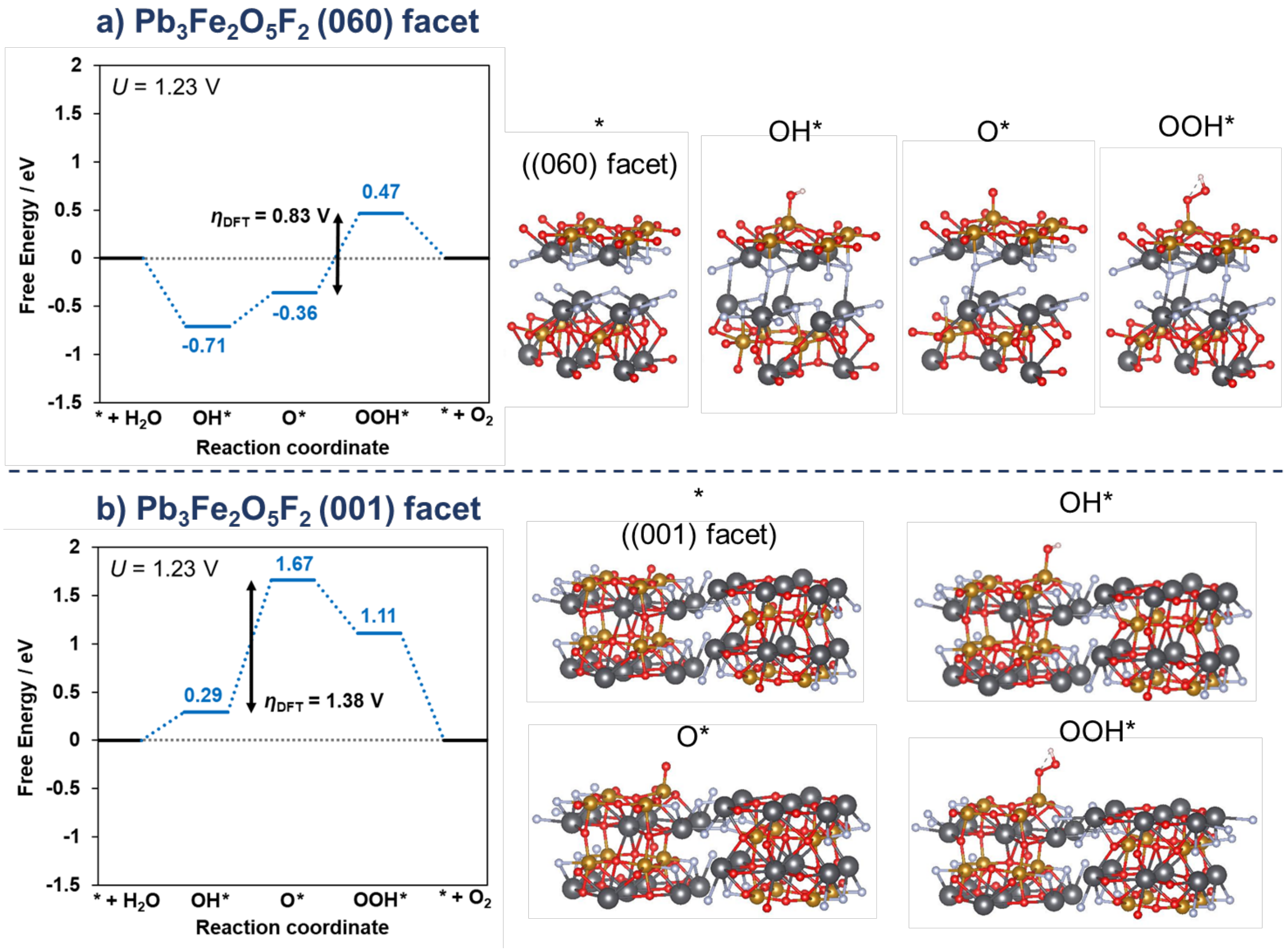

Figure S15. Gibbs energy diagrams for OER on a) (060) and b) (001) facets of $Pb_3Fe_2O_5F_2$, as obtained by DFT calculations through the AEM process, and the corresponding surface crystal structure for each state with adsorbed reaction intermediates (Pb: black, Fe: brown, O: red, F: white, H: beige). Notations and conditions except for mentioned above are the same as in Figure S13.

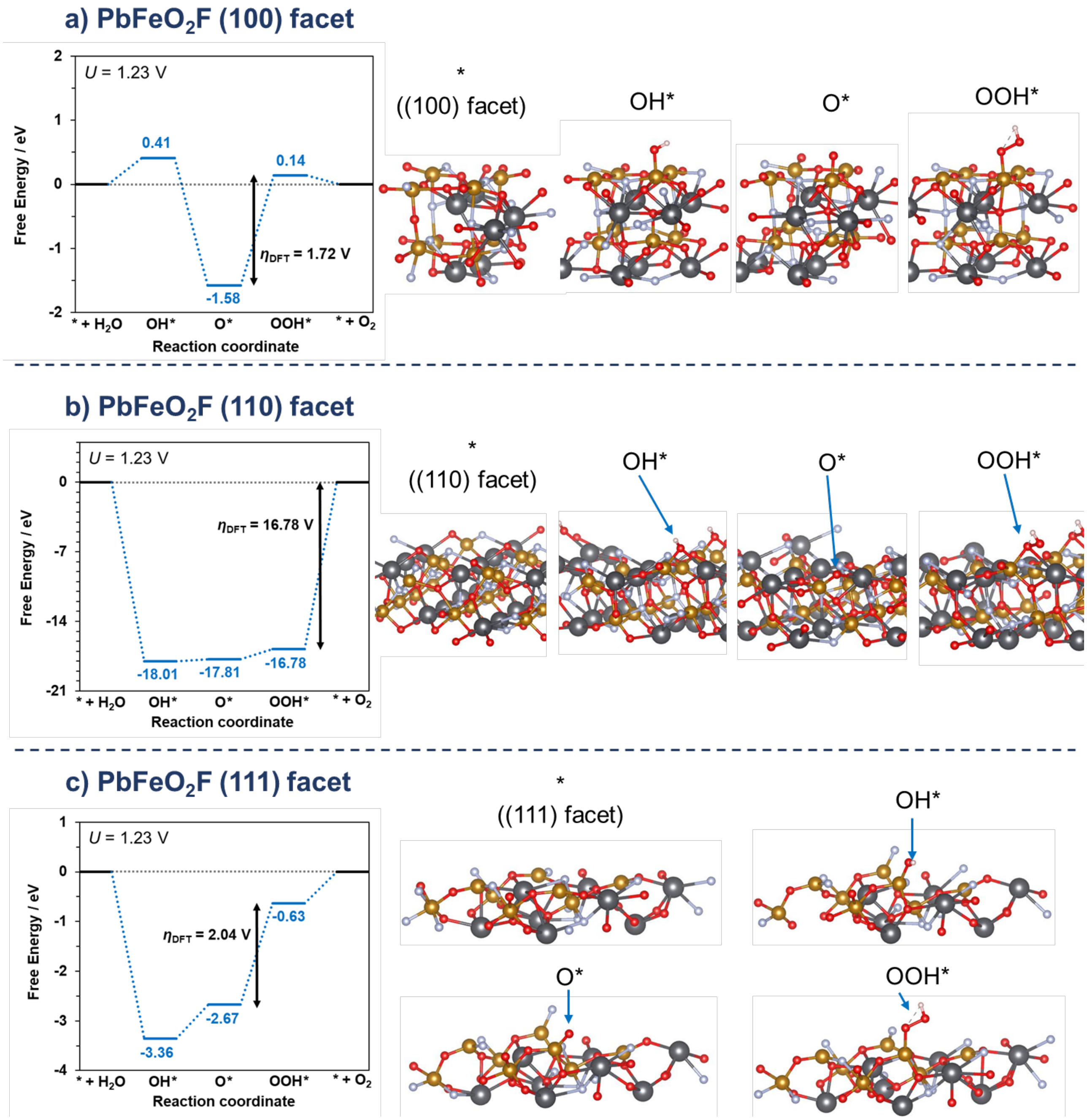

Figure S16. Gibbs energy diagrams for OER on a) (100), b) (110), and c) (111) facets of $PbFeO_2F$, as obtained by DFT calculations through the AEM process, and corresponding surface crystal structure for each state with adsorbed reaction intermediates (Pb: black, Fe: brown, O: red, F: white, H: beige). Notations and conditions except for mentioned above are the same as in Figure S13.

Table S1. Electrochemical measurement results for electrocatalysts in this study and those previously reported for Fe-based electrocatalysts[a]

Catalyst	Overpotential at 10 mA cm^{-2}_{geo} [V]	Overpotential at 0.05 mA cm^{-2}_{BET} [V]	Tafel slope [mV dec^{-1}]	Reference
$Pb_3Fe_2O_5F_2$	0.49	0.34	45	This work
$PbFeO_2F$	0.52	0.36	54	This work
α-Fe_2O_3 (99.9%, Wako)	0.66	0.39	60	This work
α-Fe_2O_3	—	0.38 ± 0.06 (at 0.5 mA)	40 ± 8	Ref S6
a-Fe_2O_3[b]	—	0.32 ± 0.02 (at 0.5 mA)	40 ± 4	Ref S6
$BiFeO_3$	0.38 (at 3 mA)	—	263	Ref S7
$LaFeO_3$	—	0.36	~57	Ref S8
$SrFeO_3$	0.40 ± 0.0065	—	66 ± 5.9	Ref S9
$CaFeO_3$	—	0.34	46	Ref S10
$CaFe_2O_4$	0.35 ± 0.0065	—	50 ± 4.4	Ref S9
$CaCu_3Fe_4O_{12}$	—	0.26	40	Ref S10
$Co_3Sb_4O_6F_6$	—	0.33	—	Ref 33
NiFeOF (15 at% Fe)	0.295	0.207	38	Ref 35
$NiFe_2F_{4.4}O_{1.8}$	0.27 ± 0.025	—	46	Ref 38
$CoFe_2F_{6.6}O_{0.7}$	0.30 ± 0.010	—	42	Ref 38
$MnFeF_{4.6}O_{0.2}$	0.500 (in 0.5 M H_2SO_4)	—	195	Ref 41
$MnFe_2F_{5.8}O_{1.1}$	0.515 (in 0.5 M H_2SO_4)	—	175	Ref 41
FeVNbTiZrOF	—	0.348 ± 0.002	110.3 ± 0.1	Ref 43
Fe_2OF_4/Carbon cloth	0.326	—	170	Ref 44
Fe_2OF_4/Nickel foam	0.238	—	48	Ref 44
$Co_{0.5}Fe_{0.5}O_{0.5}F_{1.5}$	0.220	—	27	Ref 45

[a] Strongly basic solution was used in the electrochemical measurements unless otherwise mentioned. Refs S4−S8 show oxide electrocatalysts and Refs 33, 35 38 41, 44 and 45 in the main text showed oxyfluoride electrocatalysts except for those with amorphous phase or mixing some crystallites such as oxide and fluoride. [b] 'a' denotes amorphous.

Table S2. Amounts of dissolved Pb, Fe and F in 1 M KOH solution after durability tests using $Pb_3Fe_2O_5F_2$−CNT/GS electrodes with the 12 h $Pb_3Fe_2O_5F_2$ sample.

Measurement	Dissolved amount / wt%		
	Pb[b]	Fe[b]	F[c]
Immersed for 18 h	trace	trace	trace
CV for 9 h (300 cycles)[a]	1.2	3.8	trace
CV for 18 h (600 cycles)[a]	1.2	4.3	trace

[a] Conducted after immersion in the 1 M KOH electrolyte solution for 18 h. [b] Measured by ICP-OES. [c] Measured by a fluoride ion assay kit (Ponalkit®-F, Fujifilm Wako) which can detect fluoride ions in aqueous solution with an indicator chemical. Trace in the table means under quantification limits: Pb <0.2 ppm, Fe <0.1 ppm by the ICP-OES and F <0.1 ppm by the fluoride ion assay kit.

Table S3. Refined lattice parameters, unit cell volumes and sizes of crystalline domains at room temperature for the $Pb_3Fe_2O_5F_2$ samples treated at varied heating periods (4, 8, 12 and 18 h).

$Pb_3Fe_2O_5F_2$ sample	a / Å	b / Å	c / Å	b / °	V / Å^3	Crystalline domain[a] /nm
Heated for 4 h	3.9333(2)	21.5636(7)	3.9432(2)	90.07(1)	334.44(3)	58
Heated for 8 h	3.9347(2)	21.5640(6)	3.9459(2)	90.08(1)	334.80(3)	92
Heated for 12 h	3.9349(2)	21.5713(4)	3.9470(2)	90.09(1)	335.02(3)	134
Heated for 18 h	3.9344(2)	21.5676(6)	3.9465(2)	90.08(1)	334.89(3)	94

[a] The sizes of crystalline domains were estimated using the Halder–Wagner plot (Refs S11 and S12). Contribution of peak broadening due to the instrument was corrected using the XRD pattern of Si powder.

Table S4. Anisotropic growth behavior of the crystallites qualitatively estimated for three peaks 020, 001 and 100 by using the Scherrer equation with the same samples as Table S3.

$Pb_3Fe_2O_5F_2$ sample	Crystalline domain[a] / nm		
	(020)	(001)	(100)
Heated for 4 h	67	63	63
Heated for 8 h	69	75	75
Heated for 12 h	69	76	76
Heated for 18 h	69	75	75

[a] Estimated by employing Scherrer equation for 020, 001, and 100 reflections: $D = \frac{K\lambda}{B\cos\theta}$, where D is the size of crystalline domain, B is the full-width half maximum, q is the peak position, K is the Scherrer constant (K = 0.90), and λ is the wavelength of X-ray.

Table S5. Surface atomic ratios for each element to Fe for $Pb_3Fe_2O_5F_2$ samples, as measured after synthesis at different heating times (4, 8, 12, and 18 h).

$Pb_3Fe_2O_5F_2$ sample	Pb/Fe	O/Fe	F/Fe
Heated for 4 h	1.6	3.1	0.87
Heated for 8 h	1.7	3.1	0.86
Heated for 12 h	1.7	2.9	0.85
Heated for 18 h	1.5	2.8	0.85

* Calculated from the corresponding XPS peak area spectra in Figure S10.

Table S6. Formation Gibbs free energies G_f of $Pb_3Fe_2O_5F_2$ to cut at (010), (020), (040) and (060) facets.

	$Pb_3Fe_2O_5F_2$ of crystal facet			
	(010)	(020)	(040)	(060)
G_f / eV[a]	–293.783	–293.788	–293.788	–289.453

[a] Calculated by means of the same DFT calculation condition in Experimental Section of the manuscript using the supercell with total 48 atoms as the calculation unit.

Reference

S1. Inaguma, Y.; Greneche, J.-M.; Crosnier-Lopez, M.-P.; Katsumata, T.; Calage, Y.; Fourquet, J.-L. Structure and Mössbauer Studies of F−O Ordering in Antiferromagnetic Perovskite $PbFeO_2F$. *Chem. Mater.* **2005**, 17, 1386-1390.

S2. Bera, A.; Bhattacharya, A.; Tiwari, N.; Jha, S. N.; Bhattacharyya, D. Morphology, stability, and X-ray absorption spectroscopic study of iron oxide (Hematite) nanoparticles prepared by micelle nanolithography. *Surf. Sci.* **2018**, 669, 145-153.

S3. Nakada, A.; Uchiyama, T.; Kawakami, N.; Sahara, G.; Nishioka, S.; Kamata, R.; Kumagai, H.; Ishitani, O.; Uchimoto, Y.; Maeda, K. Solar Water Oxidation by a Visible-Light-Responsive Tantalum/Nitrogen-Codoped Rutile Titania Anode for Photoelectrochemical Water Splitting and Carbon Dioxide Fixation. *ChemPhotoChem* **2019**, 3, 37-45.

S4. Dollase, W. A. Correction of intensities for preferred orientation in powder diffractometry: application of the March model *J. Appl. Crystallogr.*, **1986**, 19, 267-272.

S5. Rong, X.; Parolin, J.; Kolpak, A. M. A Fundamental Relationship between Reaction Mechanism and Stability in Metal Oxide Catalysts for Oxygen Evolution, *ACS Catal.*, **2016**, 6, 1153-1158.

S6. Smith, R. D. L.; Prévot, M. S.; Fagan, R. D.; Zhang, Z.; Sedach, P. A.; Siu, M. K. J.; Trudel, S.; Berlinguette, C. P. Photochemical Route for Accessing Amorphous Metal Oxide Materials for Water Oxidation Catalysis. *Science* **2013**, 340, 60-63.

S7. Afzal, R. A.; Park, K.-Y.; Cho, S.-H.; Kim, N.-I.; Choi, S. R.; Kim, J. H.; Lim, H.-T.; Park, J.-Y. Oxygen electrode reactions of doped $BiFeO_3$ materials for low and elevated temperature fuel cell applications. *RSC Adv.* **2017**, 7, 47643-47653.

S8. Li, H.; Chen, Y.; Xi, S.; Wang, J.; Sun, S.; Sun, Y.; Du, Y.; Xu, Z. J. Degree of geometric tilting determines the activity of FeO_6 octahedra for water oxidation. *Chem. Mater.* **2018**, 30, 4313-4320.

S9. Sugawara, Y.; Kamata, K.; Ishikawa, A.; Tateyama, Y.; Yamaguchi, T. Efficient Oxygen Evolution Electrocatalysis on $CaFe_2O_4$ and Its Reaction Mechanism. *ACS Appl. Energy Mater.* **2021**, 4, 3057-3066.

S10. Yamada, I.; Takamatsu, A.; Asai, K.; Shirakawa, T.; Ohzuku, H.; Seno, A.; Uchimura, T.; Fujii, H.; Kawaguchi, S.; Wada, K. Systematic study of descriptors for oxygen evolution reaction catalysis in perovskite oxides. *J. Phys. Chem. C* **2018**, 122, 27885-27892.

S11. Halder, N. C.; Wagner, C. N. J. Separation of particle size and lattice strain in integral breadth measurements. *Acta Crystallogr.* **1966**, 20, 312-313.

S12. Halder, N. C.; Wagner, C. N. J. Analysis of the Broadening of Powder Pattern Peaks Using Variance, Integral Breadth, and Fourier Coefficients of the Line Profile. In *Advances in X-Ray Analysis*, Springer US, 1966; pp 91-102.
